# Counting cases, conserving species: addressing highly pathogenic avian influenza in wildlife

**DOI:** 10.1002/brv.70173

**Published:** 2026-05-08

**Authors:** Ulrich Knief, Sandra Bouwhuis, Anja Globig, Anne Günther, Wouter Courtens

**Affiliations:** ^1^ Evolutionary Biology and Ecology University of Freiburg, Institute of Biology I (Zoology) Hauptstr. 1 Freiburg DE‐79104 Germany; ^2^ Institute of Avian Research An der Vogelwarte 21 Wilhelmshaven DE‐26386 Germany; ^3^ Friedrich‐Loeffler‐Institut, Institute of International Animal Health/One Health, Federal Research Institute for Animal Health Südufer 10 Greifswald‐Insel Riems DE‐17493 Germany; ^4^ Friedrich‐Loeffler‐Institut, Institute of Diagnostic Virology, Federal Research Institute for Animal Health Südufer 10 Greifswald‐Insel Riems DE‐17493 Germany; ^5^ Research Institute for Nature and Forest Havenlaan 88, bus 73 Brussels 1000 Belgium

**Keywords:** HPAI, H5N1, wildlife, wild birds, ecology, mass mortality, disease outbreak

## Abstract

Highly pathogenic avian influenza (HPAI) has become a critical threat to wildlife, shifting from a seasonal epizootic to a persistent, year‐round panzootic with global consequences. Here, we summarise the origin, evolutionary mechanisms, and expanding host range of the current H5N1 virus (clade 2.3.4.4b) and assess its impact on wildlife. Over the past 5 years, HPAI has caused the deaths of millions of wild birds, causing dramatic population declines in several seabird species. Mortality records, however, are often anecdotal, focus on localised mass die‐offs, and thus represent only a fraction of the true mortality. This lack of quantitative data limits the ability to predict outbreak dynamics and mitigate long‐term consequences. Using the northwestern European Sandwich tern (*Thalasseus sandvicensis*) population as a case study, we demonstrate the value of integrating mortality data with ecological, serological and genetic data before, during and after an outbreak. This approach uncovered age‐specific vulnerability, selective mortality, and population immunological responses, and provided insights into how breeding density, carcass removal, and host adaptation modulate outbreak dynamics. The absence of a centralised and standardised wildlife mortality monitoring framework, on the other hand, remains a major barrier to effective outbreak forecasting and conservation planning. We argue that integrating population and mortality monitoring, serological assays, and genetic analyses within a One Health framework is essential to enable early detection, targeted mitigation, and robust evaluation of outbreak impacts, and caution that without a proactive and data‐driven approach to conservation, HPAI will continue to threaten global wildlife populations, with cascading ecological, economic and public health consequences.

## I. INTRODUCTION

The highly pathogenic avian influenza (HPAI) virus subtype H5N1 belonging to clade 2.3.4.4b has made global headlines, causing the deaths of hundreds of millions of domestic poultry and wild animals (Clough, 2022; Weston, 2024; Hirschinger *et al*., 2025). Since 2021, its enzootic circulation in Europe (Pohlmann *et al*., 2022), expansion into new host species (Klaassen & Wille, 2023), and transcontinental spread to the Americas (Caliendo *et al*., 2022) and Antarctica (Bennett‐Laso *et al*., 2024; Ogrzewalska *et al*., 2024) have left, and continue to leave, a trail of ecological and economic devastation around the world.

The designation of ‘highly pathogenic’ avian influenza is based on standardised criteria in chickens (including disease severity, systemic replication and, for H5/H7 viruses, the presence of a multibasic cleavage site in haemagglutinin; WOAH, 2021) and does not necessarily generalise to severe disease in other avian species or in mammals. Nevertheless, HPAI, commonly referred to as ‘bird flu’, is highly contagious and often manifests with neurological disorders and leads to high mortality in susceptible avian hosts. In recent years, HPAI viruses of the H5N1 subtype belonging to clade 2.3.4.4b have spilled over into a broad range of hosts, including wild birds, wild terrestrial and marine mammals, fur animals, pets, and even mammalian livestock.

These developments have raised concern across disciplines, from conservation biology to public and human health (Kuiken & Cromie, 2022; Wille & Barr, 2022; Atkinson & Baillie, 2024, 2025; Nature Medicine Editorial, 2024; Runstadler, 2024; Webby & Uyeki, 2024; Lambertucci, Santangeli & Plaza, 2025). Yet, wildlife is still often viewed primarily as a disease vector, rather than as a casualty of infection or a sentinel for broader ecosystem health (Kuiken & Cromie, 2022; Klaassen & Wille, 2023; Lambertucci *et al*., 2025). As a result, and despite increasingly frequent reports of mass mortality in wild birds, the population‐level consequences for wild species remain poorly quantified (Clough, 2022; Klaassen & Wille, 2023). Most countries lack the infrastructure for systematic wildlife mortality surveillance, and available records tend to be event‐based, spatially biased, and focused on large, conspicuous species or breeding colonies.

In this review, we bring together evidence from ecological, virological, and epidemiological viewpoints to assess the consequences of HPAI outbreaks for wild birds and other wildlife. We first outline the evolutionary history and molecular characteristics of the HPAI virus H5N1 of clade 2.3.4.4b, before synthesising current knowledge on outbreak dynamics, host range expansion, and spatiotemporal trends. We then present a detailed case study of the Sandwich tern (*Thalasseus sandvicensis*) in northwestern Europe, where demographic, serological, and genomic data collected across multiple years provide unique insights into mortality patterns, immune responses, and apparent changes in host susceptibility. From this system, we derive more general conclusions regarding ecological risk factors, host traits, and population‐level impacts and evaluate how viral evolution, host ecology, and management interventions interact to shape outbreak trajectories. Finally, we stress the importance of monitoring wildlife mortality, outline priorities for enhancing such efforts, and present a framework for incorporating wildlife into One Health preparedness.

For clarity, we hereafter use ‘HPAI H5N1 virus’ as shorthand for the genetically diverse group of highly pathogenic H5N1 viruses belonging to clade 2.3.4.4b (see Section II), and ‘HPAI’ when referring to the associated disease and its ecological or demographic consequences.

## II. ORIGIN AND PROPERTIES OF THE HIGHLY PATHOGENIC H5 AVIAN INFLUENZA VIRUS GOOSE/GUANGDONG 1996

Until the 21st century, HPAI outbreaks were rare, even in poultry (Alexander & Brown, 2009). In general, HPAI viruses emerge from low pathogenic avian influenza (LPAI) viruses of the H5 or H7 subtypes through mutations in the haemagglutinin (HA) gene that shift infection from being largely restricted to intestinal and respiratory epithelial tissues to systemic viral replication in chickens (Bellido‐Martín *et al*., 2026). LPAI viruses typically cause subclinical infections or mild disease and have long been circulating in wild and captive bird populations. In 95% of the documented cases, mutations converting LPAI into HPAI viruses have occurred in environments with high bird densities, such as intensive poultry farms, where avian influenza viruses (AIVs) can easily replicate in susceptible hosts (Dhingra *et al*., 2018). Subsequently, the virus spilled over into populations of (migratory) wild birds and moved across the globe (Liu *et al*., 2005). As such, human agricultural practices are largely responsible for the current panzootic (Kuiken & Cromie, 2022).

A defining characteristic of AIVs is their RNA‐based genome, which is divided into eight segments. RNA mutates more readily than DNA, and AIVs evolve at high substitution rates, with some viral gene segments accumulating changes more rapidly in avian than in mammalian hosts (Worobey, Han & Rambaut, 2014). Substitution rates tend to be higher in poultry than in wild birds, likely reflecting differences in transmission dynamics, immune selection, and host ecology (Fourment & Holmes, 2015). When a host is co‐infected with two genetically distinct AIV subtypes, gene segments can additionally exchange through a process called reassortment, generating new virus variants with altered properties, and thereby driving the extraordinary variability and continuous evolution of AIVs (Dhingra *et al*., 2018). These changes may enable AIVs to evade host immune responses, through processes known as antigenic drift and antigenic shift (Webster *et al*., 1992; Luczo & Spackman, 2024).

Although AIVs frequently reassort and exchange genetic material, viral classification as low or highly pathogenic is determined primarily by the HA gene. Among HPAI H5N1 viruses, however, host‐specific disease severity can vary substantially between viral strains that share the same HA but differ in other gene segments (Piesche *et al*., 2024). The evolutionary history of the HA gene nevertheless remains central for phylogenetic classification, and its genetic divergence forms the basis for defining viral clades (Smith & Donis, 2015). In this nomenclature, higher‐order clades (e.g. 2.3.4) are subdivided into successively nested clades (e.g. 2.3.4.4), each representing a monophyletic lineage.

The currently dominating HPAI viruses carry an H5 HA gene and descend from a lineage first isolated as an H5N1 virus from domestic geese in Guangdong, China, in 1996, later designated as Goose (Gs)/Guangdong (Gd)/1/1996 (Alexander & Brown, 2009). Initially confined to poultry in southeast China, it was detected in wild birds for the first time in 2003, and subsequently spread to waterbirds in multiple countries across Southeast Asia (Alexander, 2007). Following its detection in wild migratory birds at Qinghai Lake in northwestern China in 2005 (Chen *et al*., 2005; Liu *et al*., 2005), the virus—later designated as clade 2.2 (Ducatez *et al*., 2017)—spread rapidly westward, reaching Europe, the Middle East, and Africa by 2006, with some evidence that wild migratory waterbirds contributed to this rapid spread (Salzberg *et al*., 2007; Global Consortium for H5N8 and Related Influenza Viruses, 2016; Lee *et al*., 2016; Shi *et al*., 2023).

After this expansion, outbreaks in wild birds declined markedly, as clade 2.2 was largely eliminated in Asia and Europe within 2 years (King *et al*., 2021). In poultry in Egypt, however, it circulated for many years until being replaced by more derived clades, most notably clade 2.3.4.4b after 2016 (El‐Shesheny *et al*., 2021; Shi *et al*., 2023). Continued circulation in domestic poultry, especially in Southeast Asia, facilitated further viral evolution (Sonnberg, Webby & Webster, 2013). Around 2005, clade 2.3.4 emerged in China (de Vries *et al*., 2015), and subsequently diversified into multiple descendant clades between 2008 and 2010 (Lee *et al*., 2016).

Among these, clade 2.3.4.4 was first detected in 2010 (Global Consortium for H5N8 and Related Influenza Viruses, 2016; Xie *et al*., 2023) and later identified in multiple countries across Asia, Europe and Africa (Antigua *et al*., 2019). While earlier HPAI viruses had consistently circulated as subtype H5N1, clade 2.3.4.4 viruses are characterised by frequent reassortment, particularly of their neuraminidase (NA) gene segment. This reassortment behaviour, often described as ‘promiscuous’, led to the repeated emergence of several H5Nx combinations (N1, N2, N3, N5, N6, N8) through genetic exchange with co‐circulating LPAI viruses, which was not observed in earlier H5 clades (de Vries *et al*., 2015; Global Consortium for H5N8 and Related Influenza Viruses, 2016; Pohlmann *et al*., 2022; Nguyen *et al*., 2025). In late 2014, a 2.3.4.4 virus, later designated as clade 2.3.4.4c (Graziosi *et al*., 2024), of subtype H5N8 was detected in North America for the first time, following intercontinental spread *via* migratory birds through the Bering Strait to Alaska (Lee *et al*., 2015; Global Consortium for H5N8 and Related Influenza Viruses, 2016; Shi *et al*., 2023). There, it reassorted with endemic LPAI viruses, giving rise to novel H5N2 and H5N1 subtypes (Lee *et al*., 2016). However, its impact on wild birds remained limited, and it disappeared from North America by 2016 (Xie *et al*., 2023).

Clade 2.3.4.4b was first detected as an H5N8 subtype in domestic ducks in eastern China in 2013 and in South Korea in 2014 [Graziosi *et al*. (2024) and references therein]. A reassortant H5N8 virus of this clade, which had acquired several gene segments from co‐circulating LPAI viruses, was isolated from wild birds during a mass mortality event at Qinghai Lake in 2016 (Li *et al*., 2017). This reassortant virus marked a turning point: it spread from China to Europe in 2016 and 2017, expanded to an unparalleled geographic range, affected a broader spectrum of species, and caused the largest documented outbreak in wild birds up to that point (King *et al*., 2021; Xie *et al*., 2023). The subsequent outbreak of 2020–2021 originated from an H5N8 virus that likely evolved in Egypt, and led to the hitherto most extensive HPAI epizootic in Europe, affecting over 22 million poultry and spreading widely among wild, predominantly anseriform, birds (EFSA *et al*., 2021; Shi *et al*., 2023; King *et al*., 2024). The current (as of December 2025) HPAI virus, subtype H5N1 belonging to the 2.3.4.4b lineage, originated mid‐2020 as a reassortant of this H5N8 virus and is likely of European origin. During this reassortment, the virus exchanged its N8 NA gene segment with an N1 segment that had been circulating in European wild birds since 2019, and additionally replaced five internal gene segments (Pohlmann *et al*., 2022; Xie *et al*., 2023). The resulting HPAI H5N1 virus was first detected in the Netherlands in October 2020 (called the ‘index case’) (Lewis *et al*., 2021) and has caused the ongoing outbreak, which has surpassed all previous events in geographic scale, ecological severity, and economic impact – spreading across continents and severely affecting wild bird populations and an unprecedented range of other wild animal species worldwide (Peacock *et al*., 2025).

## III. AN OVERVIEW OF HPAI IN WILDLIFE SINCE 2021 IN EUROPE AND BEYOND

Before 2021, HPAI was a seasonal epizootic disease in Europe, primarily affecting domestic poultry and wild land‐ and waterfowl (Galloanserae), with occasional spillover of HPAI viruses into raptors feeding on infected wild birds; these were predominantly detected through passive surveillance of severe or fatal cases. Outbreaks were most pronounced in winter, such as among barnacle geese (*Branta leucopsis*) along the North Sea coast (King *et al*., 2021). Occasionally, shorebirds were affected as well, as seen in the Wadden Sea during the winter of 2020, when approximately 3,500 red knots (*Calidris canutus*) were found to have died from a reassortant HPAI H5N3 virus (King *et al*., 2024).

### (1) A shift to year‐round infections in Europe and global spread

In 2021, the situation shifted dramatically. The HPAI H5N1 virus became enzootic in Europe (Pohlmann *et al*., 2022), meaning that cases in wild birds started to occur year‐round. Initially, they became particularly intense during the breeding season, especially in colonially breeding seabirds, but also in spillover hosts such as raptors and other scavenging species (Günther *et al*., 2024). In 2022, for example, colonially breeding northern gannets (*Morus bassanus*) (Lane *et al*., 2024), Sandwich terns (Knief *et al*., 2024) and common terns (*Sterna hirundo*) (Pohlmann *et al*., 2023) suffered substantial losses while breeding across the North and Baltic Seas. While the precise mutations or reassortments of the viral genome that enabled the initial expansion of host species remain unclear (or may not have occurred at all; Webby & Uyeki, 2024), a notable reassortment event in spring 2022 involved the circulating HPAI H5N1 virus and a gull‐adapted LPAI virus, leading to the emergence of a new H5N1 genotype in northern France (EFSA *et al*., 2023c; Fusaro *et al*., 2024; Briand *et al*., 2025). This EA‐2022‐BB genotype appeared to be particularly adapted to gulls and terns, very fatal to its hosts, highly contagious, and possibly transmitted through airborne routes (Zhang *et al*., 2022; Nagy, Černíková & Sedlák, 2025). It spread among black‐headed gulls (*Chroicocephalus ridibundus*) during the winter, and in the subsequent summer affected breeding colonies of black‐headed gulls and common terns, from the coasts to the foothills of the Alps (Hjulsager *et al*., 2026; Knief *et al*., 2026).

**Fig. 1:**
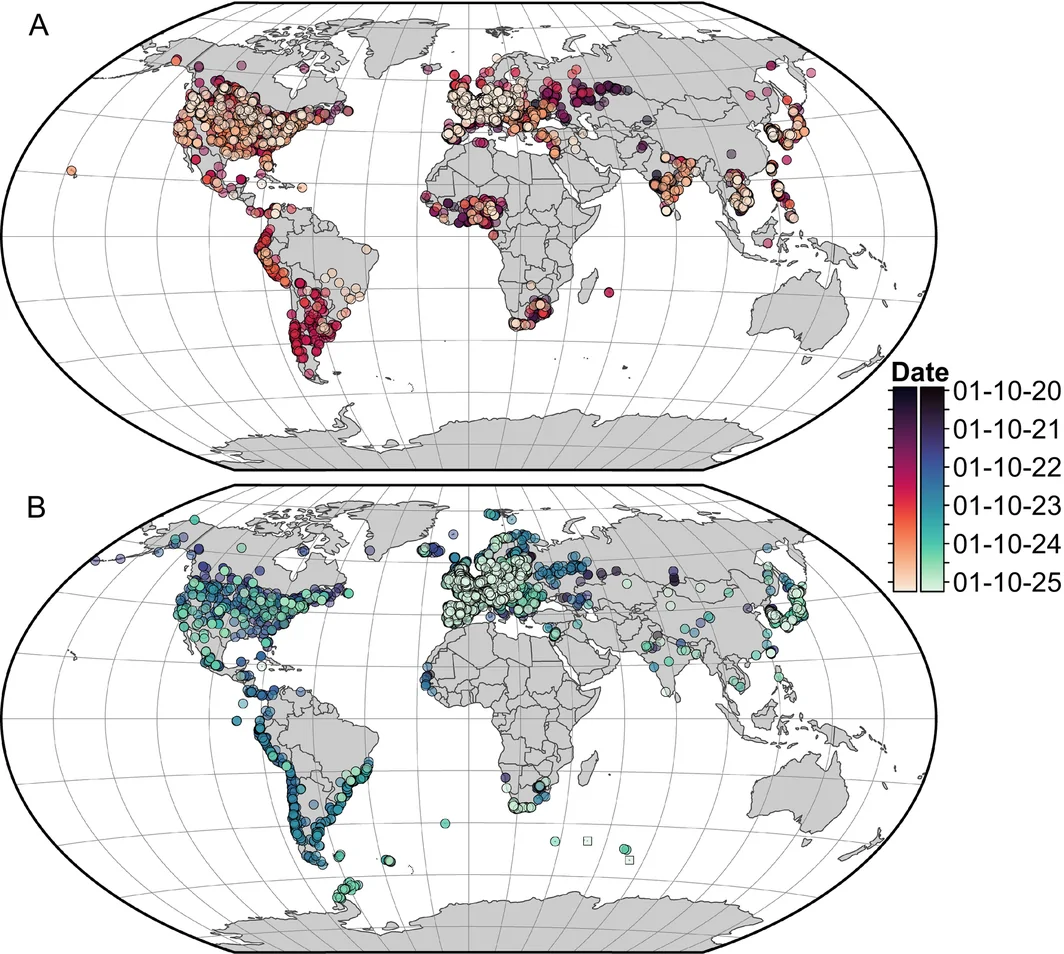
Spatiotemporal dynamics of the current HPAI H5N1 panzootic in (A) domestic (*N* = 10,460) and (B) wild (*N* = 14,350) animals. Each dot represents an avian influenza positive case from the EMPRES‐i + database (disease: ‘Influenza – Avian’) reported between 1 September 2020 and 1 December 2025. Dots may represent isolated cases or mass mortality events. Detections from Heard Island and the Crozet Islands, which are not yet included in the EMPRES‐i + database, are shown with square symbols based on published reports. The panzootic originated in Europe (index case October 2020 in the Netherlands; Lewis *et al*., 2021) and subsequently spread to Africa (December 2020; Abolnik *et al*., 2023) and East Asia (October 2021; Tian *et al*., 2023). Earlier occurrences in South and East Asia were of independent origin (Xie *et al*., 2023; Tare *et al*., 2024). North America was reached *via* two independent incursions: one from Europe (September 2021) and one from East Asia (February 2022; Giacinti *et al*., 2024; Damodaran *et al*., 2026). The virus spread from North to South America for the first time in October 2022 *via* migratory birds, with initial outbreaks in Colombia (Leguia *et al*., 2023). From there, it moved clockwise around the continent, but also overland from west to east throughout 2022–2023 (Uhart *et al*., 2024; Kuiken *et al*., 2026). The virus also reached the (Sub)Antarctic islands: the Falkland Islands in October 2023 (Banyard *et al*., 2024), South Georgia in September 2023 (Bennison *et al*., 2024), Gough and Marion Island in September 2024 (DFFE, 2025; Steinfurth *et al*., 2026), Crozet and the Kerguelen Islands in October 2024 (Clessin *et al*., 2025), and Heard Island in November 2025 (Watt & Collins, 2025). Finally, it reached Antarctica in January 2024 (Ogrzewalska *et al*., 2024). Even remote oceanic archipelagos such as the Galápagos (September 2023; Stokstad, 2023) and the Hawaiian Islands (November 2024; State of Hawai'i, 2025) have been affected. See Code1_Fig1.r for the R code used to generate this figure.

**Fig. 2:**
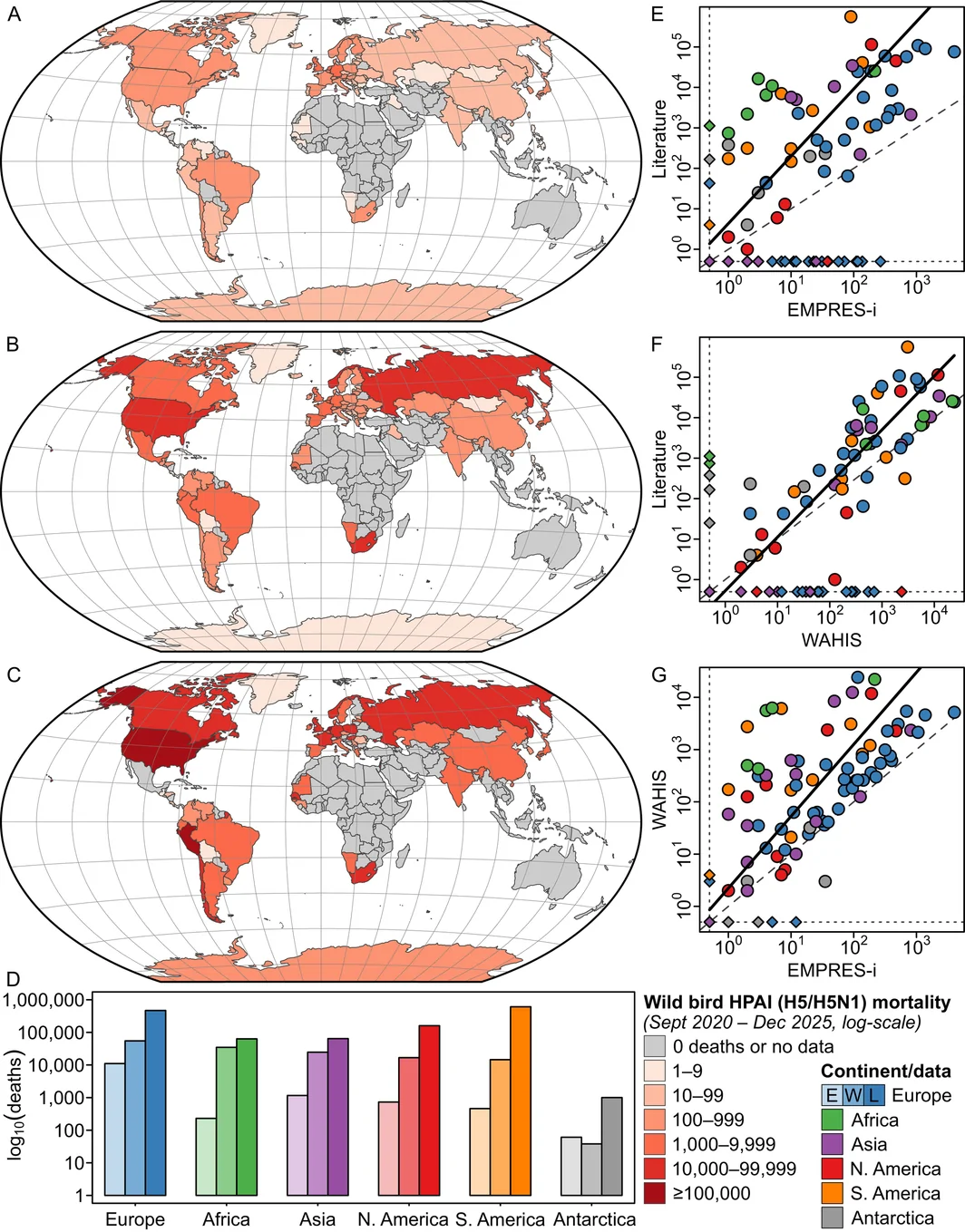
Comparison of wild bird mortality data (log‐scale) from the EMPRES‐i+ database (see Data File S3), the World Animal Health Information System (WAHIS) (see Data File S2), and our literature review (see Data File S1) of reported mass mortality events since the emergence of HPAI H5N1 of clade 2.3.4.4b in autumn 2020 until December 2025. For this comparison, the number of reported deaths per country was summed across species and age classes, as this information is often missing from databases and even from some published reports. (A–C) Number of reported deaths per country in (A) EMPRES‐i+, (B) WAHIS, and (C) the literature review. (D) Total number of reported deaths per continent (left to right: E = EMPRES‐i+, W = WAHIS, L = Literature). Overall, 13,734, 146,027, and 1,367,732 dead wild birds were reported through EMPRES‐i+, WAHIS, and the literature, respectively. (E–G) Pairwise comparisons between country‐based data sets. Diamonds along horizontal and vertical dotted lines indicate missing records in one or the other data set. These were excluded from the major axis regression. The dashed line represents the 1:1 line (perfect agreement), and the thick black line shows the major axis regression. Major axis regression slopes in E and F are significantly different from 1 (*P* = 4.2 × 10^−3^ and *P* = 6.5 × 10^−3^, respectively). Colours represent continents. See Code2_Fig2.r for the R code used to generate this figure.

Globally, the HPAI H5N1 virus associated with the current panzootic has spread to every continent except Oceania (Wille *et al*., 2024). It reached the Americas in September 2021 (Fig. 1) (Damodaran, Jaeger & Moncla, 2026), and the most severe outbreaks among wild bird populations in North America occurred in eastern Canada during 2022, with approximately 40,391 recorded deaths, primarily among northern gannets and common murre (*Uria aalge*) (Avery‐Gomm *et al*., 2024) (Fig. 2, see online Supporting Information Appendix S1 and Data Files S1–S3). Subsequently, South America faced severe impacts in 2022 and 2023, when the virus affected immunologically naïve wild bird populations with no prior exposure to HPAI. An estimated 667,000 wild birds succumbed (Uhart *et al*., 2024; Kuiken *et al*., 2026) (Fig. 2, Data Files S1–S3). Reports from Peru alone included 254,793 Guanay and Neotropical cormorants (*Leucocarbo bougainvilliorum* and *Nannopterum brasilianus*), 235,643 Peruvian boobies (*Sula variegata*), and 57,447 Peruvian pelicans (*Pelecanus thagus*), with these numbers representing up to 50% of the global populations of these species (Gamarra‐Toledo *et al*., 2023; Kuiken *et al*., 2026).

The HPAI H5N1 virus reached the (Sub)Antarctic region *via* South America in the summer of 2023, with confirmed outbreaks in the Falkland Islands and South Georgia (Banyard *et al*., 2024; Bennison *et al*., 2024). By January 2024, the virus had also been detected in Antarctica, marking the first known incursion of HPAI viruses into the continent (Bennett‐Laso *et al*., 2024; Ogrzewalska *et al*., 2024). Later that year, it spread from South America to Gough Island in the central South Atlantic Ocean, as well as to the Prince Edward, Crozet, and Kerguelen Islands – remote archipelagos in the Southern Indian Ocean. Genetic analyses confirmed this eastward transatlantic transmission route, rather than spread *via* Africa (Duvenage, 2024; Clessin *et al*., 2025; Steinfurth *et al*., 2026). In the autumn of 2024, the virus also reached Hawai'i in the Central Pacific, most likely carried by migratory birds from Alaska (State of Hawai'i, 2025), and was subsequently detected in November 2025 on Heard Island in the Southern Indian Ocean (Watt & Collins, 2025).

### (2) Beyond birds: HPAI infections in mammals

The global spread of the HPAI H5N1 virus has also led to increasing numbers of infections in wild mammals. While media attention has mostly focused on infected dairy cattle in North America (e.g. Capua & Fanelli, 2024; Nguyen *et al*., 2025), mammalian infections in the wild have been reported predominantly in predators and scavengers, which most likely contract the virus by consuming infected dead or weakened birds. Both marine mammals, such as seals, porpoises, and dolphins, as well as terrestrial ones, including foxes, bears, and mustelids, have been affected in large numbers (ENETWILD Consortium *et al*., 2024; Plaza *et al*., 2024a; Puryear & Runstadler, 2024). While outbreaks in terrestrial species mostly involved single, isolated cases, the outbreaks among marine mammals, particularly in South America, were especially severe (Webby & Uyeki, 2024). Between late 2022 and 2023, the virus spread along the coasts of Peru, Chile, Argentina, and Uruguay, eventually reaching Brazil. During this period, at least 31,894 South American sea lions (*Otaria flavescens*) (Plaza *et al*., 2024b; Uhart *et al*., 2024; Kuiken *et al*., 2026) and 17,400 southern elephant seals (*Mirounga leonina*) succumbed to the disease (Campagna *et al*., 2024; Kuiken *et al*., 2026). In Argentina's Valdés Peninsula, the outbreak led to the death of more than half of the local southern elephant seal population. Concerningly, during these mammalian outbreaks, the virus acquired mutations that enhanced its adaptation to mammals and seemingly enabled mammal‐to‐mammal transmission (Tomás *et al*., 2024; Uhart *et al*., 2024; Nguyen *et al*., 2025; Pardo‐Roa *et al*., 2025).

These quantitative wildlife data should not give the false impression that we have a comprehensive understanding of the dynamics and impact of HPAI outbreaks in wild animal populations (Klaassen & Wille, 2023; Lambertucci *et al*., 2025; Couty *et al*., 2026). In reality, they probably represent only a small fraction of what actually occurred, and a centralised system for collecting quantitative mortality data is urgently needed to estimate the full impact of HPAI (see Fig. 2). In the following section, we use data collected during the HPAI outbreaks in European Sandwich tern colonies to exemplify what we can derive from such data, and pose the questions that remain to be addressed. By drawing on additional relevant information, we try to generalise these findings to other species, and we highlight how scientific endeavours could benefit species conservation and the economy, as well as public health. Finally, we propose concrete political actions that should be taken to improve our current understanding of HPAI in wildlife.

## IV. THE CASE OF HPAI IN NORTHWESTERN EUROPEAN SANDWICH TERNS

### (1) Risk factors for Sandwich terns

Similar to many other seabirds, Sandwich terns breed in dense colonies on the ground, with up to seven nests per square metre, spaced just far enough apart to prevent neighbours from reaching one another. These colonies can host several thousand breeding pairs, but there were only 67 colonies across northwestern Europe in 2022, making Sandwich terns an ideal model species to study HPAI dynamics both at the colony and population level. The high breeding density, combined with the species' tendency to defecate around nest sites, can result in elevated viral loads within colonies and facilitate both direct bird‐to‐bird and indirect transmission *via* faecal–oral routes or environmental contact. Regular inter‐ and intra‐annual exchange of birds between colonies (Spaans, Leopold & Loos, 2021; R.S.A. van Bemmelen, personal communication) then creates a scenario in which, once the virus enters a colony and is sufficiently adapted to the host species, it can spread rapidly, potentially wiping out a substantial proportion of the entire population.

### (2) The 2022 outbreak: devastating impact

The northwestern European Sandwich tern population suffered greatly from HPAI in 2022. Knief *et al*. (2024) collected data on breeding pair numbers, mortality rates, and whether carcasses were removed from colonies. Before the outbreak, the population comprised 59,632 breeding pairs, equivalent to 119,264 adult individuals, excluding late breeders and non‐breeding birds such as prospectors. Most of the 65 colonies with HPAI status data were concentrated along the North Sea coasts of France, Belgium, the Netherlands, Germany, Denmark, and the UK. During the summer of 2022, 19,326 adult birds were found dead across 39 of the 65 monitored colonies in northwestern Europe. Based on additional data, we estimated that total mortality exceeded 17% of the breeding population, amounting to more than 20,500 adult birds. Additionally, nearly all chicks in affected colonies perished. While occasional reproductive failure is less critical for this long‐lived species, which is capable of surviving up to 32 years and adapted to low breeding success, the loss of adult birds poses a far greater threat to the population's long‐term viability (Sæther *et al*., 2016).

### (3) Trends in 2023 and beyond: fewer deaths and evidence for an immune response

During the winter of 2022–2023, reports from West Africa, where Sandwich terns overwinter, documented over 25,000 deaths among seabirds, including at least 2,394 Sandwich terns (Barlow *et al*., 2023; Ndao, 2023; Jatta *et al*., 2025).

**Fig. 3:**
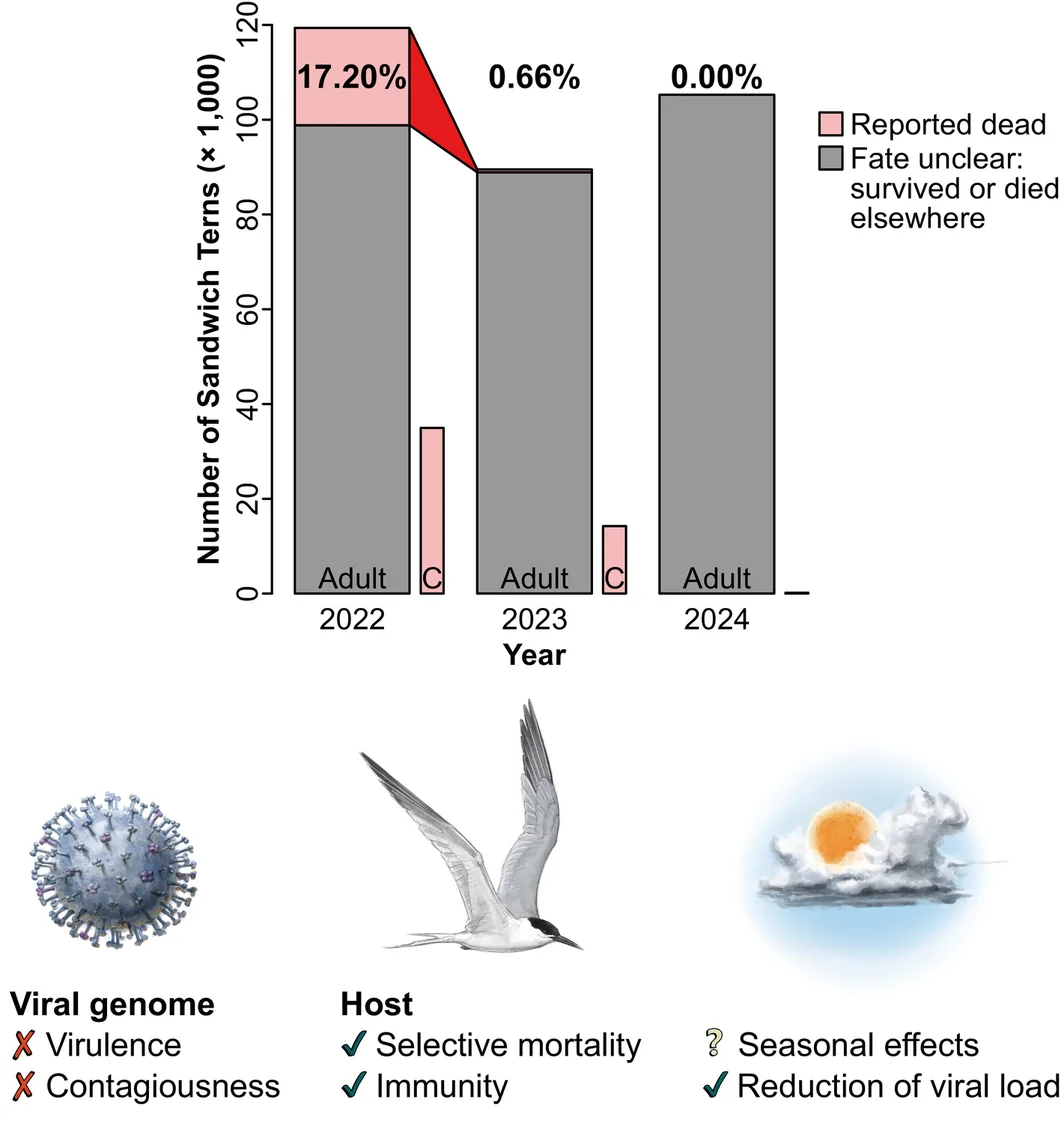
Breeding bird population size and mortality in the northwestern European Sandwich tern population during the 3 years since the HPAI H5N1 outbreak in 2022. Adult mortality is based on counts of dead birds found in colonies and along shorelines; chick mortality (C) is mostly estimated based on available reports of breeding success or estimates of chick deaths. The decline in mortality from 2022 to 2023 is highlighted, and possible explanations are illustrated below. Either the virus, the host, or the environment must have changed. Available evidence suggests that the virus did not alter its virulence or contagiousness, but rather that host populations adapted through selective mortality and the build‐up of immunity. Evidence for environmental effects is mixed: seasonal factors, such as rising temperatures or increased ultraviolet exposure, may have played a role, and environmental viral loads were likely reduced due to large‐scale carcass removal adopted in 2023, and possibly also due to lower breeding bird densities in some species (e.g. common terns). See Code3_Fig3.r for the R code used to generate this figure. Illustrations were created by Javier Lazaro.

Anticipating another devastating outbreak during the breeding season of 2023, the situation unfolded differently than expected. Breeding pair numbers fell by 25% to 44,748 pairs (Fig. 3). However, while the virus remained present in at least 32 colonies, spread to colonies in the Mediterranean Sea (Valle *et al*., 2024) and caused another near‐total loss of chicks in affected colonies, adult birds were largely spared. Only 592 adult Sandwich terns were found dead, which represents a reduction in mortality of about 96% (Fig. 3). This unexpected shift raised questions. Although carcass removal had proved to be an effective mitigation measure (Knief *et al*., 2024) and was widely implemented in 2023, this alone could not explain the difference. The virus did not appear to have decreased in virulence or contagiousness among gulls and terns. Earlier genotypes circulating in 2022 among terns and northern gannets (EA‐2021‐AB and EA‐2020‐C, respectively) were replaced in 2023 by EA‐2022‐BB, which appeared to be even more adapted to gulls and terns (Pohlmann *et al*., 2023; Fusaro *et al*., 2024; Briand *et al*., 2025). The new genotype continued to impact black‐headed gulls and common terns heavily, decimating colonies of both species across Europe. Moreover, Sandwich tern chicks still died in large numbers from EA‐2022‐BB in 2023 [all 41 genotyped individuals (EFSA *et al*., 2023b; Fusaro *et al*., 2024; A. Fusaro, personal communication)]. So why did adult mortality decline so dramatically?

Several factors may be at play. First, populations may have partially acquired population‐level immunity through seroconversion (development of detectable antibodies following infection or vaccination) of previously immunologically naïve adults surviving the initial outbreak. Around 20% of adult breeding Sandwich terns in Germany tested positive for antibodies against H5Nx in both 2023 and 2024 (*N* = 50 in 2023 and *N* = 60 in 2024). However, it remains unclear whether these antibodies are neutralising and whether they were present in and prior to 2022, as birds with antibodies were still found to die from the disease in a common tern population in northern Germany (*S. Bouwhuis*, personal observation). Nonetheless, these findings raise the possibility that an increased level of immunity helped protect affected populations from mass mortality. Consistently, common terns that were severely affected in both 2022 and 2023 in northern Germany showed a marked increase in H5Nx antibody prevalence, from less than 20% to around 40% between 2023 and 2024 (Knief *et al*., 2026).

Additionally, the most susceptible individuals may have been removed from the population during the initial outbreak. Analyses of 28 years of Belgian and Dutch bird ringing and recovery data (1995–2022: 43,000 unique recoveries from over 50,000 ringed birds) revealed that age classes were not equally affected by HPAI. Specifically, older birds appeared to suffer the highest mortality (Courtens *et al*., 2025). This suggests that, by 2023, after the majority of the population had been exposed to the virus (Rijks *et al*., 2022), the most vulnerable individuals may already have been removed from the population or substantially reduced in number due to selective mortality (i.e. natural selection). Standing genetic variation at (host‐specific) disease resilience genes may harbour adaptive variants that can rapidly rise in allele frequency under such extreme selection pressure (Boonyanuwat *et al*., 2006; Drobik‐Czwarno *et al*., 2018; Chen *et al*., 2025). As a result, the individuals most susceptible to severe disease would no longer be present in the population and could neither act as amplifier hosts nor contribute to subsequent mortality counts. Together, both immunity and selective mortality may have enhanced the protection of the remaining northwestern European Sandwich tern population.

Irrespective of the mechanism, the reduced adult mortality provided cautious optimism for population recovery, further reinforced by the absence of reported mass mortality events in Africa during the winter of 2023–2024. The specific virus variant (genotype EA‐2022‐BB), which caused severe mortality in black‐headed gulls and common terns during the 2023 breeding season, disappeared in September 2023 (Fusaro *et al*., 2024). It was replaced by variants that seem to be less adapted to gulls and terns. In line with this, the breeding season of 2024 saw no noticeable infections or mortality among adult Sandwich terns or their chicks, nor among other colonial breeding birds that had been heavily affected in previous years, such as black‐headed gulls and common terns. The northwestern European Sandwich tern population remained at a relatively low level, but increased substantially to 52,634 breeding pairs (Fig. 3). This sudden rise may reflect the recruitment of previously non‐breeding or younger individuals filling vacant breeding sites. These individuals may represent a ‘silent reserve’ within the population. However, this reserve may now be largely depleted, suggesting that further recovery toward pre‐HPAI population levels is likely to be slow and may take decades.

## V. GENERAL PATTERNS IN HPAI OUTBREAK DYNAMICS AMONG WILD BIRD SPECIES

### (1) Anticipated spread, unpredictable impact

The global spread of HPAI viruses has been anticipated and discussed among researchers since their expansion from Asia in 2005 (Butler & Ruttimann, 2006), especially with regard to their potential arrival in South America and Antarctica (Global Consortium for H5N8 and Related Influenza Viruses, 2016; Hurt *et al*., 2016). Rather than being unexpected, the scale of the current panzootic aligns with these earlier warnings. However, outbreaks appear idiosyncratic, meaning that it is next to impossible to predict which species will be affected next, where and when outbreaks will occur, or how severe they will be. This unpredictability may arise because HPAI H5N1 transmission appears to be driven by interconnected transmission networks involving multiple sympatric species (Knief *et al*., 2026; Matthiopoulos *et al*., 2025). Consequently, continuous monitoring and data collection remain essential (Caliendo *et al*., 2024; Nguyen *et al*., 2025).

### (2) Varying impact across species, populations, and individuals

The impact of HPAI varies considerably among species. For example, it has been devastating for the great skua (*Stercorarius skua*), a scavenging seabird species, with more than half of the global population lost (Tremlett *et al*., 2024). Some South American and South African seabird species suffered substantial losses as well, accounting for up to 50% of their world populations (Abolnik *et al*., 2023; Kuiken *et al*., 2026). Northern gannets were affected throughout their global distribution range in 2022 (Avery‐Gomm *et al*., 2024; Lane *et al*., 2024), and robust model‐based mortality estimates for this species indicate losses of around 33% of the European population (Matthiopoulos *et al*., 2025), suggesting that global mortality approached or exceeded 25% of the breeding population (Walker *et al*., 2025). Arguably, the most accurate data come from northwestern European Sandwich terns, where, as described above, mortality exceeded 17% of the breeding bird population in 2022 (Knief *et al*., 2024), and a 25% loss in breeding pairs in 2023. By contrast, great cormorants (*Phalacrocorax carbo*) in the Baltic Sea region were subject to limited outbreaks, which had little to no effect on population size (Bregnballe *et al*., 2024), and some species may not exhibit clinical signs when infected with HPAI viruses at all, thereby serving as reservoirs. This includes mallards (*Anas platyrhynchos*), some other dabbling ducks (Gaidet *et al*., 2008; Koethe *et al*., 2020; Teitelbaum *et al*., 2023), and possibly also larger gull species (*Larus* spp.) (Arnal *et al*., 2015; Tarasiuk *et al*., 2022).

Even closely related species may differ in their susceptibility to the disease. While mortality among adult bald eagles (*Haliaeetus leucocephalus*) in North America was high, adults of the closely related European white‐tailed eagle (*Haliaeetus albicilla*) were not affected in Germany, despite likely being exposed to the virus, as indicated by the HPAI‐related death of their chicks during the beginning of the enzootic in Europe (Nemeth *et al*., 2023; Günther *et al*., 2024). In Norway, however, some adult white‐tailed eagles were affected (Bøe *et al*., 2024), suggesting potential regional differences in susceptibility or exposure. Beyond these inter‐ and intraspecific differences, mortality rates also varied considerably among populations of other species. For example, during the 2022 breeding season in Germany, mortality ranged from 0% to nearly 50% in different common tern colonies and from 1% to over 60% in Sandwich tern colonies (Pohlmann *et al*., 2023).

Beyond these inter‐ and intraspecific patterns, there is also substantial variation among individuals within populations in their response to HPAI virus exposure. Not all infections are lethal (Harvey *et al*., 2025; Hirschinger *et al*., 2025; Knief *et al*., 2026; McLaughlin *et al*., 2025; Ponchon *et al*., 2026), and subclinical infections may remain undetected while still altering movement behaviour, which could either amplify or constrain transmission (Duriez *et al*., 2023; Careen *et al*., 2024; Jeglinski *et al*., 2024). Such individual‐level variation provides a plausible explanation for why populations with similar exposure histories can experience markedly different mortality rates, and why infection and transmission may persist even where observed mortality is low.

### (3) Key factors influencing HPAI dynamics

From this variation across species, populations, and individuals, we can distil some factors influencing the dynamics of HPAI outbreaks that seem to be general across species:

(*i*) Breeding bird density appears to be an important factor influencing both the risk and severity of outbreaks, as observed in northern gannets (Lane *et al*., 2024), common terns (Marchowski *et al*., 2024), and great cormorants (Bregnballe *et al*., 2024). Species‐specific behaviours may further affect transmission risk. For example, defecation within breeding colonies by Sandwich terns (Cullen, 1960), kleptoparasitism of gulls, frigatebirds, skuas, and terns (García *et al*., 2020; Gorta *et al*., 2024), or the use of communal club and bathing sites by great skuas, referred to as ‘ponds of death’ due to their association with repeated mass mortality events (Camphuysen & Gear, 2022; Bregnballe, Meise & Packmor, 2023), may facilitate large‐scale outbreaks. Common terns have also been observed attacking sick, abnormally behaving conspecifics (*S. Bouwhuis*, personal observations), which may also contribute to viral spread.

(*ii*) HPAI H5N1 outbreaks in northern gannets (Matthiopoulos *et al*., 2025) and common terns (Ewing & Bouwhuis, 2025) show that within‐colony transmission can be highly efficient, with estimated basic reproductive numbers (*R*_0_) exceeding 2 and comparable to, or in some cases exceeding, those reported for poultry systems (Kirkeby & Ward, 2022), underscoring the importance of conspecific bird‐to‐bird transmission for rapid local amplification in dense seabird colonies.

(*iii*) Transmission between colonies appears to be shaped by colony connectivity (Boulinier, 2023; Jeglinski *et al*., 2024) and processes that extend beyond single species. Matthiopoulos *et al*. (2025) documented pronounced temporal synchrony in HPAI outbreaks across European northern gannet colonies, such that outbreaks occurred nearly simultaneously across distant sites. This synchrony could not be explained by independent within‐colony outbreak dynamics or short‐range, within‐species inter‐colony transmission alone, but rather suggested shared latent processes acting across the colony network. Such epidemiological connectivity may be established through multispecies transmission or movements of non‐breeding conspecifics, as also suggested for black‐headed gulls and common terns (Knief *et al*., 2026).

(*iv*) Carcass removal can be an effective mitigation measure to reduce further spread of the virus (Knief *et al*., 2024). However, its success likely depends on early outbreak detection. Otherwise, it could potentially even have the opposite effect at the population level, if infected birds leave the colony in response to human disturbance and thereby spread the virus to other breeding sites.

(*v*) Instead of the virus altering its virulence or contagiousness, or the environment undergoing substantial changes, it appears that host populations may adjust to the virus over time through individual‐level responses (phenotypic plasticity, adaptive immunity) and population‐level processes (senescence‐driven mortality, natural selection). The build‐up of immunity and selective mortality of the most vulnerable individuals, i.e. those that previously acted as amplifier hosts, may reduce exposure and provide greater protection for the remaining individuals (Fig. 3).

(*vi*) Age‐related mortality can arise from multiple, potentially interacting factors. In Sandwich terns, immunosenescence (Bartleson *et al*., 2021) and age‐dependent variation in breeding propensity may both contribute to age‐related mortality (Courtens *et al*., 2025). Similar patterns have been observed in common terns, where differences in age and phenology are linked to differential mortality risk (*S. Bouwhuis*, personal observation). In addition, genetic predisposition may contribute to selective mortality, favouring individuals with stronger immunity, or greater resistance or tolerance to infection (Boonyanuwat *et al*., 2006; Drobik‐Czwarno *et al*., 2018; Chen *et al*., 2021; Chen *et al*., 2025). Further genomic studies in host species are needed to understand better the genetic basis of host‐specific disease resilience and selective survival in wild birds.

(*vii*) Improved population‐level protection may be reflected by the observation that subsequent mass mortality events appear to become less likely after an initial severe outbreak. For instance, the outbreaks with the most profound impact in North America occurred in eastern Canada in 2022, resulting in the death of over 40,000 wild birds (Avery‐Gomm *et al*., 2024). By 2023, the mortality rate in the region had dropped by 93%, and the few HPAI outbreaks that did occur primarily affected different species than in the previous year (Cormier *et al*., 2024). Similarly, observed mortality rates were 42% lower in 2023 compared to 2022 in common terns (Bouwhuis, 2025), and no major outbreaks or mass mortalities were reported in adult northern gannets (Cormier *et al*., 2024; Tyndall *et al*., 2024) or Sandwich terns in 2023. In South America, following the peak in mortality in 2022 and 2023, seabird mass mortality events were rarely reported in 2024 and 2025 (Uhart & Vanstreels, 2025). The fact that large numbers of Sandwich tern chicks died in 2023 may further suggest that adults were protected by acquired immunity, a hypothesis that fits with observations in mute swans (*Cygnus olor*) during an outbreak of HPAI in the UK in 2008 (Pybus *et al*., 2012), and that should be validated through further longitudinal seroprevalence studies.

### (4) A glimpse of hope? Demographic buffering and compensatory dynamics

While the immediate impacts of HPAI have often been catastrophic, some demographic processes may allow partial recovery, at least in the short term, provided that populations retain sufficient numbers of individuals and are not subjected to repeated outbreaks or additional anthropogenic threats. Seabirds, for example, typically exhibit delayed maturation, spending their early years away from the breeding grounds (Hamer, Schreiber & Burger, 2001). These young birds could be considered a ‘silent reserve’, as they appear to fill vacant breeding sites – a pattern observed in common murres, northern gannets, and common terns in 2023 and 2024 (Birkhead & Hatchwell, 2025; Sceviour, Ward & Wilhelm, 2025; *S. Bouwhuis*, personal observation), and that also seems to apply to Sandwich terns. Furthermore, following severe HPAI‐related adult mortality, a smaller colony size can lower intraspecific aggression and competition for food, leading to reduced foraging effort and higher breeding success among survivors. Such density‐dependent compensation has been documented in northern gannets, where breeding success rebounded sharply in the year following mass mortality and individual foraging trips covered smaller areas, were shorter in distance, and of reduced duration (Ponchon *et al*., 2026), consistent with classical seabird population theory (Ashmole, 1963).

## VI. RELEVANCE OF QUANTITATIVE MORTALITY DATA COLLECTION FOR NATURE AND SPECIES CONSERVATION

In today's world, wildlife faces numerous threats, such as habitat loss, climate change and pollution, leading to a largely human‐induced sixth mass extinction (Ceballos *et al*., 2015). Seabirds are the world's most threatened group of birds (Paleczny *et al*., 2015) with many species facing various additional threats, including fisheries, tourism, and the introduction of predators to previously suitable breeding sites, all contributing to their population declines worldwide (Phillips, Fort & Dias, 2023). The recent HPAI outbreaks must now be added to this growing list of threats. If HPAI continues to recur in seabird populations, it could rapidly become devastating for some species.

### (1) Implications for bird monitoring programs

Due to their sensitivity, widespread distribution, and ease of observation, birds serve as prime bioindicators, offering insights into environmental conditions across different trophic levels. Consequently, numerous monitoring programs track avian population trends and investigate the causes of population changes. Without comprehensive mortality data from HPAI outbreaks, sudden population declines would remain unexplained. Such data are therefore vital and their collection must be maintained, even during acute disease outbreaks.

When combined with further monitoring efforts, long‐term population studies facilitate more in‐depth epidemiological analyses. For instance, the analysis of selective age‐related mortality in Sandwich terns was only possible due to extensive and continuous ringing efforts across Europe, which provided a robust foundation for survival modelling. Such efforts were initially aimed at understanding the natural history of the species, but have proven invaluable for addressing emerging conservation challenges (Johnston *et al*., 2025). They should, therefore, receive greater support and funding (also see Clutton‐Brock & Sheldon, 2010). A recent study on peregrine falcons (*Falco peregrinus*), a spillover host, similarly illustrates what can be gained from long‐term ringing and recovery data: reduced adult survival during the HPAI period in the Netherlands in 2022 was inferred despite limited direct mortality observations, and demographic resilience analyses revealed long‐lasting population‐level effects (Badia‐Boher *et al*., 2026). The accuracy and precision of such demographic inferences, however, depend on species‐specific characteristics, particularly mobility and detectability, which differ markedly between territorial raptors and highly mobile colonial seabirds.

Where sufficiently resolved mortality or incidence time series are available within colonies, basic reproductive numbers (*R*_0_) can be estimated to describe colony outbreaks (Ewing & Bouwhuis, 2025; Matthiopoulos *et al*., 2025). Such estimates provide a compact measure of transmission intensity and allow comparisons across colonies, years, and virus genotypes, as well as evaluation of mitigation measures, such as carcass removal or density management. Importantly, however, *R*_0_ estimation requires precisely the kind of standardised, time‐resolved quantitative field data that are currently missing for most outbreaks in wildlife, reinforcing the need for coordinated mortality monitoring.

Beyond local transmission intensity, time‐resolved mortality data collected across multiple colonies enable inference on large‐scale outbreak dynamics. Such analyses allow estimation of key epidemiological parameters at the intra‐colony level, reconstruction of transmission pathways between colonies, and inference on overall mortality, even where carcass recovery is incomplete (Matthiopoulos *et al*., 2025). This illustrates how coordinated mortality monitoring expands the range of epidemiological questions that can be addressed.

### (2) Implications for conservation

In the case of Sandwich terns, quantitative mortality data played a crucial role in prompting the German Lower Saxony Wadden Sea National Park administration and the German Federal Agency for Nature Conservation (BfN), together with the Federal Ministry for the Environment, Nature Conservation, Nuclear Safety and Consumer Protection (BMUV), to initiate a species conservation program. A key focus of this program is the establishment of effective preventive measures. These include an early warning system, currently implemented through video surveillance, and, in one colony, active surveillance. As soon as an abnormally behaving or dead bird is detected, testing can be initiated, and measures can be taken to contain the infection. Active surveillance, i.e. monitoring the prevalence of HPAI viruses in Sandwich terns, is carried out by capturing breeding birds and testing them for the presence of viral RNA using molecular methods. Such measures should also be implemented for other species that have suffered from recent HPAI outbreaks.

As breeding bird density is a key factor influencing both the risk and severity of outbreaks, reducing densities or the number of birds in a colony could lower infection risk and limit outbreak sizes. One potential strategy is the creation or restoration of natural breeding habitats. While this may be challenging for gregarious species such as Sandwich terns (although still possible, for example through the expansion of suitable protected areas), it may be more readily achievable for other species. For instance, common terns readily nest on floating platforms (e.g. Manikowska‐Ślepowrońska, Ślepowroński & Jakubas, 2022), and increasing the number of these platforms could help distribute birds more evenly, thereby potentially reducing individual densities and the risk of disease transmission through reduced contact rates (Ewing & Bouwhuis, 2025).

### (3) Implications for vaccination

Vaccination against HPAI viruses has so far been considered only in a small number of wild bird species. Among seabirds, vaccination trials have been conducted in African penguins (*Spheniscus demersus*) and king penguins (*Aptenodytes patagonicus*). In African penguins, a controlled trial involving 24 captive individuals tested different vaccine platforms, including a HA‐based virus‐like particle vaccine and a conventional inactivated vaccine, using vaccination strategies with and without booster doses (Roberts *et al*., 2024). Immune responses varied depending on vaccine type and protocol. Booster vaccination increased and prolonged antibody titres, whereas a single dose induced detectable antibody responses, although titres declined over time in most individuals and persisted beyond 1 year only in a single bird.

In king penguins, vaccination using a messenger RNA (mRNA)‐based H5 vaccine induced robust antibody responses that appeared to be more persistent over time (Lejeune *et al*., 2026). Evidence from other long‐lived seabirds further suggests that vaccine‐induced antibody responses can persist for several years: experimental vaccination against Newcastle disease virus resulted in long‐lasting antibody titres and maternal transfer in procellariiform seabirds (Ramos *et al*., 2014). A recent zoo‐based vaccination trial demonstrated that an RNA replicon‐based H5 vaccine can induce durable neutralising antibody responses after two doses in a range of avian taxa, including seabirds (Stettler *et al*., 2025). Together, these findings indicate that vaccine‐induced immunity in seabirds can be long‐lasting, but is likely dependent on species, vaccine type, and vaccination protocol.

In a different conservation context, vaccination was also implemented as an emergency intervention in California condors (*Gymnogyps californianus*) in the USA. After 21 of the remaining 561 individuals died from HPAI in early 2023, surviving birds were vaccinated under strict regulations. By October 2024, nearly half of the population (*N* = 207) had received at least one dose, and no further casualties were reported among them (U.S. Fish & Wildlife Service, 2024).

For a species like the Sandwich tern, which is fortunately still relatively common, vaccination at the population level remains impractical and politically undesired. Moreover, as RNA viruses with segmented genomes, HPAI viruses evolve rapidly through mutation and reassortment, facilitating the emergence of antigenically novel variants that may escape vaccine‐induced immunity. Any vaccination‐based strategy would therefore require repeated re‐vaccination and extensive surveillance of wild bird populations to detect and monitor potential escape mutants.

### (4) Early detection and response

Since HPAI viruses cannot be eradicated in wild populations, management efforts must, at least at present, focus on early detection and rapid response rather than prevention. Vaccination may offer a solution for selected, highly endangered species, but it likely remains impractical for most wild birds, particularly in the absence of non‐invasive, passively delivered vaccination strategies that are capable of inducing sterilising immunity. Likewise, controlling virus entry points seems infeasible given the complexity of transmission pathways. Given these constraints, the most effective strategy is to improve how we monitor diseases and respond to outbreaks, which hinges on robust, quantitative mortality data. Without such data, long‐term conservation measures will remain dangerously inadequate.

## VII. IMPROVING QUANTITATIVE MORTALITY DATA COLLECTION IN WILD ANIMALS

### (1) The need for systematic wildlife mortality monitoring

Current surveillance efforts in wild birds primarily focus on detecting the presence of the virus (EMPRES‐i+), mostly by means of opportunistic sampling of sick or dead individuals in areas with high human density. Although informative, such qualitative data yield only limited understanding of the full extent of mortality and its long‐term consequences for affected populations (Fig. 2). To move beyond a reactive crisis response, we need a supportive, systematic and standardised approach to collecting quantitative mortality data, combined with diagnostics and genomic epidemiology (Table 1; Klaassen & Wille, 2023; Atkinson & Baillie, 2025). Coordinated bird and disease monitoring programs, improved data‐sharing frameworks, and closer integration of ecological and epidemiological research are essential. These efforts would not only help quantify impacts more accurately, but also enable the development of an effective and robust early warning system for future outbreaks (Couty *et al*., 2026). The Pathogen Harmonized Observatory (PHAROS) database (pharos.viralemergence.org) is a promising global initiative, but still in its infancy (Schwantes *et al*., 2025).

**Table 1:** Overview of monitoring parameters for assessing the impact and dynamics of HPAI in seabird colonies. Parameters are classified by data type, invasiveness, time investment, major spatial inference scale (colony scale), and whether they provide a snapshot or longitudinal insight. Invasiveness and time investment were assessed qualitatively based on field experience and expert judgement. The final column summarises the primary value or application of each parameter in the context of HPAI surveillance. Parameters flagged with directly support mitigation measures during HPAI outbreaks. Data type: = demographic or trait‐based monitoring; = diagnostics/virological testing; = genetic or immunological insights; = remote sensing/automated monitoring. Colony scale: Within = intracolony‐level understanding; Between = intercolony‐level understanding; Both = intra‐ and intercolony‐level understanding. Temporal insight: Snapshot = one‐time or point‐in‐time data; Longitudinal = repeated measures needed.

Parameter	Data type	Invasiveness (1–5)	Time investment (1–5)	Colony scale	Temporal insight	Value
Species affected	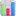	1	1	Between	Snapshot	Species range and vulnerability, also includes associated carnivores and other mammalian species
Dead adults count	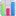	1	2	Both	Snapshot	Adult mortality estimation
Dead chicks count	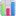	1	2	Both	Snapshot	Chick mortality estimation
Reporting ringed birds	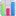	1	2	Between	Longitudinal	Survival modelling, age‐biased mortality
Collection of dead birds 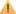	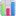	2	3	Within	Snapshot	Sex and morphometric patterns in mortality, reduces transmission risk
Onset of infection	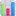	2	3	Between	Longitudinal	Spatiotemporal spread, epidemiological mapping
Breeding success	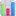	2	4	Within	Longitudinal	Impact of infection on reproductive outcome
Longitudinal within‐season mortality	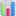	3	4	Within	Longitudinal	Fatality curves over time, estimation of basic reproductive number (*R*_0_)
Environmental sampling (guano/soil/water)	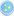	1	2	Within	Snapshot	Detection of viral presence in environment
Rapid test on dead birds in remote areas 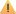	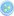	2	2	Within	Snapshot	Fast virus detection, triggers carcass removal or containment
PCR test on dead birds 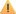	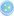	2	3	Within	Snapshot	Confirmation of viral presence, triggers biosecurity responses
Virus patho‐ and genotyping	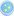	2	4	Within	Snapshot	Characterisation of avian influenza virus
Virus whole‐genome sequencing	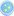	2	5	Both	Longitudinal	Genomic epidemiology, establish connections between outbreak events
Swab (live sick birds)	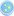	4	4	Within	Snapshot	Viral shedding detection
Swab (live healthy birds)	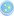	4	4	Within	Snapshot	Asymptomatic carriers, reservoirs
Host genetic sampling (live or dead)	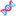	3	3	Both	Snapshot	Susceptibility/resistance genotypes
Blood/serum (sick birds)	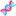	5	4	Within	Snapshot	Seroconversion, immune response during infection
Blood/serum (healthy birds)	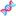	5	4	Within	Snapshot	Seroprevalence, past exposure, potential reservoirs
Longitudinal antibody titres	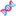	5	5	Within	Longitudinal	Duration of immunity in survivors
Camera or drone surveillance 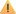	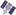	1	3	Within	Longitudinal	Early detection of behavioural change, carcass appearance, colony collapse onset, triggers early mitigation
GPS tracking	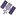	5	4	Between	Longitudinal	Movement patterns, risk assessment of viral spread

GPS, global positioning system; PCR, polymerase chain reaction.

For poultry, retrospective mortality data are accessible through systems such as animal disease insurance funds. By contrast, wildlife mortality data collection has been largely *ad hoc*, depending on private initiatives, existing records and contributions of local conservation managers, who themselves are not evenly distributed across wildlife populations. Until now, there has been no centralised system for systematically tracking wildlife mortality. However, the unprecedented scale of HPAI cases in wild birds since 2022 has made efforts to create such a system increasingly urgent.

A dedicated institution or program for systematic wildlife mortality monitoring would need to coordinate with relevant authorities, train personnel in biosafety and biosecurity, and develop standardised protocols for field data collection. Additionally, such an institution could expand and coordinate active surveillance, an approach increasingly recognised as essential by the European Union and global health organisations, such as the World Health Organization (WHO), the Food and Agriculture Organization of the UN (FAO), and the World Organization for Animal Health (WOAH).

The World Animal Health Information System (WAHIS) of the WOAH collects data on epidemiologically important diseases in domestic and wild animals, including HPAI (Cãceres *et al*., 2023). It is the most comprehensive—and currently the only—global source of HPAI‐related case numbers in wild animals (Klaassen & Wille, 2023). However, the database is incomplete: for quality assurance, notifications must be submitted by officially recognised reference laboratories, which precludes reporting from many regions and research programmes, even when outbreaks are well documented. In addition to numerous outbreaks being missed, existing entries are often inconsistent and lack essential details. Reports frequently group multiple species within a single outbreak and do not specify whether adults or chicks were affected, making it impossible to quantify species‐specific impacts reliably.

To illustrate the extent to which current reporting systems underestimate wildlife mortality, we compared reports of wild bird deaths from three sources: the EMPRES‐i+ database, WAHIS, and our comprehensive literature review of reported mass mortality events since the emergence of HPAI H5N1 of clade 2.3.4.4b in the autumn of 2020 (Fig. 2). For this comparison, reported deaths were aggregated per country across species and age classes, as such information is frequently missing or inconsistently reported in EMPRES‐i+ and WAHIS. Despite broadly similar geographic patterns, total reported mortality differed strikingly among data sources, with 13,734, 146,027, and 1,367,732 dead wild birds recorded in EMPRES‐i+, WAHIS, and the literature, respectively. Pairwise comparisons at the country level revealed systematic underreporting in both official databases relative to the literature, with major axis regression slopes significantly deviating from unity (Fig. 2E,F). Although our analysis focuses on wild birds, reported HPAI‐related mortality in wild mammals showed a similar pattern of underreporting across data sources (EMPRES‐i+: 616, WAHIS: 300, literature: 58,022), underscoring that this limitation extends beyond avian hosts. At the same time, WAHIS and EMPRES‐i+ include reports from a larger number of countries than the published literature, reflecting their broad global acceptance and mandatory reporting structures. This likely indicates that many smaller or less‐conspicuous outbreaks are documented administratively, but never enter the scientific literature, which tends to focus on particularly severe, unusual, or conservation‐relevant events. Together, these patterns underscore that official databases and the scientific literature provide complementary but incomplete perspectives, reinforcing the need for integrated systems that combine broad geographic coverage with quantitative detail.

Some regional systems do exist though: in the USA, the Wildlife Health Information Sharing Partnership (WHISPers) collects quantitative information on wildlife mortality events, but it suffers from the same lack of detail as the WAHIS database. In the UK, a smartphone app (Epicollect5) has been developed to streamline data entry and reporting. In response to the arrival of HPAI viruses in the (Sub)Antarctic region, the Scientific Committee on Antarctic Research (SCAR) has proposed innovative surveillance strategies and established an HPAI database to compile all suspected and confirmed outbreaks in the area, with the aim of monitoring the spread of the virus and understanding better its impacts on wildlife (Wille *et al*., 2025).

### (2) Reduction of regulatory barriers to active surveillance

Passive surveillance, based on the reporting and investigation of sick or dead animals, remains the most effective method for early detection of HPAI outbreaks. Active surveillance, by contrast, involves the targeted sampling of live birds and is typically aimed at detecting active virus infections through swabs while also allowing assessment of clinical condition in captured individuals. While active surveillance can provide valuable information, particularly for research and risk assessment, samples from live birds often yield low detection rates and require substantial logistical efforts and financial resources.

Serological surveillance addresses a complementary and, in some contexts, more integrative question by capturing evidence of past exposure and population‐level immunity, particularly when implemented as long‐term surveillance. Because antibodies persist beyond the short window of active infection, serology can provide insights into cumulative exposure with fewer samples than would be required for virus detection alone (e.g. McLaughlin *et al*., 2025). Despite this, serological data remain even scarcer than virus detection data, in part because serological sampling requires blood collection from live birds, which is more invasive than swab‐based virus detection and therefore subject to stricter animal welfare regulations and approval processes [but see Giacinti *et al*. (2025) and McLaughlin *et al*. (2025) for recent work on minimally invasive sampling approaches]. This regulatory asymmetry has led to a systematic underrepresentation of serological data in active surveillance, highlighting the need for surveillance frameworks in which serology is integrated as a core component rather than an optional add‐on.

In many countries, rigid and time‐consuming animal welfare approval processes hinder both responsive and proactive surveillance. These constraints are often compounded by additional permitting requirements, including access permissions for protected areas, capture and ringing permits, and associated reporting obligations. Such bureaucratic hurdles can make timely investigations almost impossible, especially given the unpredictable nature of HPAI outbreaks and the uncertainty about which species will be affected next. By contrast, more flexible regulatory frameworks enable scientists and authorities to respond more rapidly and effectively.

### (3) Balancing data collection with biosafety and biosecurity

Data collection during active outbreaks is essential for understanding transmission dynamics and for assessing population‐level impacts. Delayed access to affected sites, whether due to regulations, logistics, institutional hesitancy, or concerns over welfare, can result in the loss of critical information. While the need to minimise disturbance and ensure biosafety is paramount, these goals can be met through the use of personal protective equipment (PPE), strict field protocols, and collaboration with veterinary authorities (Bregnballe *et al*., 2023; Nature Medicine Editorial, 2024). To improve outbreak responses, site managers and government administrations must find a workable balance between data collection needs and animal welfare and human health and safety.

### (4) Bridging the gap between field and laboratory data

Quantitative field data and qualitative laboratory data must be better integrated. The two data sources are generated using different methodologies, techniques, and risk assessments, which can create challenges in combining them effectively. Differences in sampling effort, detection probabilities, and diagnostic sensitivities further complicate direct comparisons. Inconsistent communication and limited mutual understanding between field and laboratory teams can also result in mismatched expectations and data gaps. Addressing these challenges requires standardised protocols and interdisciplinary collaboration. Building stronger networks and fostering mutual trust between ecologists, epidemiologists, and laboratory researchers will be essential to ensuring responsible data collection and accurate risk assessments. This could be realised by regular subject‐specific cross‐sectoral meetings and feedback loops, interdisciplinary workshops, shared digital platforms and databases, and long‐term institutional partnerships formalised through memoranda of understanding (MoUs) between academic institutions, public health agencies and governmental institutions.

### (5) Increasing funding and institutional support

As the global spread of HPAI is likely to continue, delaying the establishment of structured monitoring systems will only weaken the ability to respond to future outbreaks. Yet, securing funding for interdisciplinary research remains a major hurdle.

Despite the urgency resulting from recent outbreaks, researchers and institutions attempting to implement integrated surveillance combining ecological analysis, genetic research, and field‐based data collection have often struggled to secure adequate support. Proposals are frequently deemed insufficiently ‘medical’, not focused enough on direct species conservation, or not interdisciplinary in the narrow ways defined by funding agencies. These recurring obstacles point to a deeper issue: the importance of integrated wildlife mortality data collection is still not fully recognised within existing funding frameworks.

This mindset must change. Rigid frameworks and isolated thinking are no longer fit for purpose in the face of complex health challenges. New concepts and approaches have already been proposed (e.g. Kuiken, 2021), but their implementation remains slow. Without more flexible funding mechanisms and stronger institutional support, our capacity to respond to future emerging disease outbreaks will remain reactive rather than proactive, which places both wildlife and public health at continued risk. The European Wildlife Disease Association (EWDA) is trying to address this by promoting transformative research in wildlife health. However, meaningful progress requires a stronger and sustained commitment from national governments and funding agencies (Krammer *et al*., 2025). Only with coordinated investment in long‐term infrastructure, workforce development, and cross‐sectoral research can we build a truly resilient One Health approach.

## VIII. QUANTITATIVE WILDLIFE MORTALITY DATA ARE ESSENTIAL FOR THE ONE HEALTH APPROACH TO HPAI

Effectively containing HPAI requires a comprehensive understanding of the interplay between the virus, the environment, and its hosts. A One Health approach (Harvey *et al*., 2023; Bertram *et al*., 2024; Bellido‐Martín *et al*., 2026) is essential, one that explicitly integrates wildlife into surveillance and response efforts, even as poultry farming remains the primary economic focus.

### (1) HPAI transmission is a two‐way street

The transmission of HPAI viruses between wild birds and poultry is not unidirectional. While poultry farms can act as the source of infection, wild birds can also introduce the virus into commercial flocks and even mammalian populations (Kuiken & Cromie, 2022; Pardo‐Roa *et al*., 2025; Signore *et al*., 2025; Damodaran *et al*., 2026). For example, the HPAI H5N1 virus of clade 2.3.4.4b reached North America via wild birds from Iceland and Greenland (Caliendo *et al*., 2022; Günther *et al*., 2022; Harvey *et al*., 2023; Ahrens *et al*., 2024), subsequently spread across North and South America largely through wild bird movements (Pardo‐Roa *et al*., 2025; Damodaran *et al*., 2026), and later arrived in Hawai'i *via* migratory birds travelling from Alaska along the Pacific flyway (State of Hawai'i, 2025). Moreover, reports from the European Food Safety Authority (EFSA) document numerous instances of wild birds transmitting the virus to both poultry and mammalian farms across Europe.

When large numbers of wild birds become infected, the viral load in the environment rises, increasing the risk of transmission not only to poultry, but also to mammals, including humans. Understanding and managing HPAI in wildlife is therefore not just a matter of species conservation, it has far‐reaching economic and public health implications.

### (2) Ethical responsibility

Humans have played a direct role not only in causing the decline of many species, but also in the emergence of HPAI viruses, as these viruses evolved in intensive poultry farming (Dhingra *et al*., 2018). Wild birds therefore are not the cause of this crisis. They are its victims. Some species may never fully recover from these outbreaks. A critical step in addressing this crisis is the systematic collection of quantitative wildlife mortality data. Such data are vital for moving from reactive, short‐term crisis management to proactive evidence‐based prevention, conservation and long‐term containment strategies. Ultimately, it is our ethical responsibility to act, not only to conserve wildlife and maintain ecosystem stability, but also to safeguard human health and prevent future zoonotic threats.

## IX. CONCLUSIONS

(1) The emergence and global spread of HPAI H5N1 of clade 2.3.4.4b in wild bird populations has exposed critical gaps in our understanding of the dynamics and impacts of HPAI outbreaks in wild animal populations. While the devastating consequences are qualitatively evident, we remain unable to provide accurate and precise assessments of the ecological impacts for most species and ecosystems. This shortcoming arises because the currently established global HPAI reporting systems were not designed with wildlife in mind. Although they provide valuable information, these general‐purpose disease surveillance tools are primarily intended for domestic livestock. As a result, they are poorly suited to capture the scale, species‐specific impacts, and ecological consequences of HPAI outbreaks in wild populations.
(2) We urgently need dedicated and systematic wildlife mortality monitoring, which includes innovative surveillance strategies for remote regions, streamlined data entry solutions, and open‐access data sharing. Such a system must integrate mortality data with diagnostic and viral genetic data, which typically become available at different times during an outbreak. The database established by the Scientific Committee on Antarctic Research for monitoring HPAI in Antarctic wildlife may serve as a promising model. Combined with, for example, smartphone‐based reporting tools, such a system could set a new gold standard for global wildlife mortality data collection.
(3) Addressing the data deficiency is not merely a scientific necessity, but an ethical imperative. Wild birds are an integral part of the current HPAI panzootic, but they are not its origin. They are suffering the consequences of a crisis largely driven by human agricultural practices. Even with limited data and resources, research during the current panzootic has yielded important insights into viral evolution, host adaptation, and outbreak mitigation in wildlife. Critically, it has also revealed that host species characteristics, such as immunity, genetic background, behaviour, and ecology, play a fundamental role in shaping outbreak dynamics and outcomes.
(4) What we now need is recognition that HPAI in wildlife is not only a conservation issue threatening biodiversity, but also a serious concern for public health and food security. We call for political commitment to support ecological research as an integral part of safeguarding against the current and future zoonotic threats.

## AUTHOR CONTRIBUTIONS

Conceptualisation and visualisations: U.K. Writing: U.K. prepared the original draft. The final manuscript incorporated input from all authors, and all authors approved the final version before submission.

## Supporting information


**Appendix S1.** Description of data queries and processing, and reference list for Data File S1.

**File S1.** Quantitative HPAI H5N1‐related mortality data in wild animals, extracted from literature published between September 2020 and December 2025.

**File S2.** HPAI‐related mortality data in wild animals, extracted from the WAHIS database on 29 January 2026.

**File S3.** HPAI‐related mortality data in wild and domestic animals, extracted from the EMPRES‐i+ database on 6 January 2026.

**Figure S1.** R code used to construct Fig. 1.

**Figure S2.** R code used to construct Fig. 2.

**Figure S3.** R code used to construct Fig. 3.

## Data Availability

The data that supports the findings of this study are available in the supplementary material of this article.
