# Supplementary material for: Counting cases, conserving species: addressing highly pathogenic avian influenza in wildlife
Source: Biol Rev Camb Philos Soc. 2026 May 8;101(5):2190–213. doi: 10.1002/brv.70173 (PMC13542358; doi:10.1002/brv.70173)
Supplement: Supplementary file 1 [file BRV-101-2190-s006.docx]

**Avian influenza in wildlife: counting cases, conserving species**

**Ulrich Knief^1,*^, Sandra Bouwhuis^2^, Anja Globig^3^, Anne Günther^4^ and Wouter Courtens^5^**

^1^ University of Freiburg, Institute of Biology I (Zoology), Evolutionary Biology and Ecology, Hauptstr. 1, DE-79104 Freiburg, Germany. Orcid: 0000-0001-6959-3033

^2^ Institute of Avian Research, An der Vogelwarte 21, D-26386 Wilhelmshaven, Germany. Orcid: 0000-0003-4023-1578

^3^ Friedrich-Loeffler-Institut, Institute of International Animal Health/One Health, Federal Research Institute for Animal Health, Südufer 10, DE-17493 Greifswald-Insel Riems, Germany. Orcid: 0009-0006-1966-6760

^4^ Friedrich-Loeffler-Institut, Institute of Diagnostic Virology, Federal Research Institute for Animal Health, Südufer 10, DE-17493 Greifswald-Insel Riems, Germany. Orcid: 0000-0001-5176-6346

^5^ Research Institute for Nature and Forest, Havenlaan 88, bus 73, 1000 Brussels, Belgium. Orcid: 0000-0002-5806-0343

^*^ Author for correspondence (E-mail: [ulrich.knief@biologie.uni-freiburg.de](mailto:ulrich.knief@biologie.uni-freiburg.de); Tel.: 0049-761-203-2911)

**Appendix S1. Description of data queries and processing, and reference list for Data File S1.**

**EMPRES-i+ Data Query and Preparation**

We extracted data from the FAO Emergency Prevention System – Global Animal Disease Information System (EMPRES-i+) database (<https://empres-i.apps.fao.org/diseases>) on 6 January 2026 using the following filters:

- Disease: ‘Influenza – Avian’
- Observation date range: 01/01/2002 – 06/01/2026

For all analyses, we restricted records to events with an observation date or, if unavailable, a report date between 01/09/2020 and 31/12/2025. We further subsetted the data to records with diagnosis status classified as ‘confirmed’.

To focus on the current HPAI panzootic, we retained only records with serotype information indicating ‘H5N1 HPAI’ or ‘H5 HPAI’. For records from Antarctic regions, where subtype information was occasionally less specific, we additionally retained entries classified as ‘HPAI’.

Based on the species information provided, we classified events as affecting either birds or mammals.

We used the region information to assign events to continents. Central American outbreaks were grouped with North America, and all Subantarctic islands were assigned to Antarctica.

**WAHIS Data Query and Preparation**

We extracted data from the World Animal Health Information System (WAHIS) database (<https://wahis.woah.org/#/dashboards/qd-dashboard>) on 29 January 2026 using the following filters:

- Disease:

‘Avian influenza A viruses of high pathogenicity (Inf. with) (bovines [Bos taurus]) (2025-)’,

‘High pathogenicity avian influenza viruses (Inf. with) (poultry)’,

‘Influenza A virus (Inf. with)’,

‘Influenza A viruses of high pathogenicity (Inf. with) (non-poultry including wild birds) (2017-)’

For all analyses, we restricted records to events that occurred between 2020 and 2025 and excluded records reported for the ‘Jan–Jun 2020’ semester.

To focus on the current HPAI panzootic, we retained only records with serotype information indicating ‘H5N1’, ‘H5 (N untyped)’, ‘H5 (N untyped);H5N1’, ‘not typed’, or ‘pending’.

Based on the species information provided, we classified events as affecting either birds or mammals.

We used the region information to assign events to continents. Central American outbreaks were grouped with North America, and all Subantarctic islands were assigned to Antarctica.

Some countries, such as the USA, report only the number of HPAI-positive wild bird cases without specifying mortality in WAHIS. To account for this and other missing values, we inferred the number of deaths by subtracting the number of animals reported as culled or slaughtered from the total number of cases, and set remaining missing values to zero where appropriate.

WAHIS reports occasionally include duplicate entries for a single outbreak, particularly where both generic (e.g. H5) and specific (e.g. H5N1) subtypes are listed separately. In such cases, we retained the record with the more specific subtype designation and removed exact duplicates.

**Literature Data Query and Preparation**

Since the onset of HPAI outbreaks in seabirds, we compiled a literature database of reports on infections and mortality in wild animals. In addition to peer-reviewed articles, this database included web pages, press releases, and other forms of grey literature. All sources were screened for quantitative information on wildlife mortality, and reference lists were examined for additional relevant reports. We further reviewed all European Food Safety Authority (EFSA) reports, which summarize major mass mortality events worldwide. In addition, we conducted targeted literature searches using *Google Scholar* with the search terms (‘HPAI’ OR ‘avian influenza’) AND ‘mass mortality’.

For the USA, we additionally extracted data from the Wildlife Health Information Sharing Partnership Event Reporting System (WHISPers) on 6 January 2026 using the following filters:

- Event type: ‘Mortality/Morbidity’
- Date range: 1/1/2021 – 31/12/2025
- Diagnosis type: ‘Virus’
- Event diagnosis:

‘Highly Pathogenic Avian Influenza (AI virus H5)’,

‘Highly Pathogenic Avian Influenza (AI virus H5N1)’,

‘Highly Pathogenic Avian Influenza (AI virus, not otherwise specified)’

- Animal type:

‘Bird’,

‘Mammal’

We integrated the WHISPers data into our literature database, ensuring that duplicate entries were removed. All identified events are listed in Data File S1.

Based on the species information provided, we classified events as affecting either birds or mammals.

We used the reported region information to assign events to continents. Central American outbreaks were grouped with North America, and all Subantarctic islands were assigned to Antarctica.

**Analyses**

All analyses were conducted in R (v4.5.1; R Core Team, 2025). Country polygons were obtained from Natural Earth using the rnaturalearth (v1.1.0; Massicotte & South, 2025) package and handled as simple features using sf (v1.0-21; Pebesma & Bivand, 2023). Country names were harmonised across data sets by manual recoding and matched to Natural Earth country names prior to mapping. All spatial data were projected to a Winkel Tripel coordinate reference system.

Agreement between data sources was assessed using major axis regression on log_10_-transformed country-level mortality counts, implemented in smatr (v3.4-8; Warton *et al.*, 2012).

**References**

Massicotte, P. & South, A. (2025). rnaturalearth: world map data from Natural Earth, https://CRAN.R-project.org/package=rnaturalearth.

Pebesma, E. & Bivand, R. (2023). *Spatial data science: with applications in R*. Chapman and Hall/CRC, Boca Raton, FL.

R Core Team. (2025). R: a language and environment for statistical computing. R Foundation for Statistical Computing, Vienna, Austria.

Warton, D. I., Duursma, R. A., Falster, D. S. & Taskinen, S. (2012). smatr 3 – an R package for estimation and inference about allometric lines. *Methods in Ecology and Evolution* **3**, 257–259.

**References for Data File S1**

Abolnik, C., Phiri, T., Peyrot, B., de Beer, R., Snyman, A., Roberts, D., Ludynia, K., Jordaan, F., Maartens, M., Ismail, Z., Strydom, C., van der Zel, G., Anthony, J., Daniell, N., De Boni, L., Grewar, J., Olivier, A. & Roberts, L. (2023). The molecular epidemiology of clade 2.3.4.4b H5N1 high pathogenicity avian influenza in Southern Africa, 2021–2022. *Viruses* **15**, e1383.

Abolnik, C., Roberts, L. C., Strydom, C., Snyman, A. & Roberts, D. G. (2024). Outbreaks of H5N1 high pathogenicity avian influenza in South Africa in 2023 were caused by two distinct sub-genotypes of clade 2.3.4.4b viruses. *Viruses* **16**, e896.

Alexandrou, O., Malakou, M. & Catsadorakis, G. (2022). The impact of avian influenza 2022 on Dalmatian pelicans was the worst ever wildlife disaster in Greece. *Oryx* **56**, 813–813.

Avery-Gomm, S., Barychka, T., English, M., Ronconi, R. A., Wilhelm, S. I., Rail, J.-F., Cormier, T., Beaumont, M., Bowser, C., Burt, T. V., Collins, S. M., Duffy, S., Giacinti, J. A., Gilliland, S., Giroux, J.-F., Gjerdrum, C., Guillemette, M., Hargan, K. E., Jones, M., Kennedy, A., Kusalik, L., Lair, S., Lang, A., Lavoie, R. A., Lepage, C., McPhail, G., Montevecchi, W. A., Parsons, G. J., Provencher, J. F., Rahman, I., Robertson, G. J., Seyer, Y., Soos, C., Ward, C. R. E., Wells, R. & Wight, J. (2024). Wild bird mass mortalities in eastern Canada associated with the highly pathogenic avian influenza A(H5N1) virus, 2022. *Ecosphere* **15**, e4980.

Ballmann, M. Z. & Lilipaly, S. J. (2024). *Wild bird mortality in the Dutch Delta in 2023*. Deltamilieu Projecten, Vlissingen, the Netherlands.

Banyard, A. C., Begeman, L., Black, J., Breed, A. C., Dewar, M., Fijn, R. C., Kuiken, T., Serafini, P. P., Uhart, M. M., Vanstreels, R. E. T. & Wille, M. (2023). *Continued expansion of high pathogenicity avian influenza H5 in wildlife in South America and incursions into the Antarctic region*. OFFLU Statement.

Banyard, A. C., Bennison, A., Byrne, A. M. P., Reid, S. M., Lynton-Jenkins, J. G., Mollett, B., De Silva, D., Peers-Dent, J., Finlayson, K., Hall, R., Blockley, F., Blyth, M., Falchieri, M., Fowler, Z., Fitzcharles, E. M., Brown, I. H. & James, J. (2024). Detection and spread of high pathogenicity avian influenza virus H5N1 in the Antarctic region. *Nature Communications* **15**, e7433.

Banyard, A. C., Lean, F. Z. X., Robinson, C., Howie, F., Tyler, G., Nisbet, C., Seekings, J., Meyer, S., Whittard, E., Ashpitel, H. F., Bas, M., Byrne, A. M. P., Lewis, T., James, J., Stephan, L., Lewis, N. S., Brown, I. H., Hansen, R. D. E. & Reid, S. M. (2022). Detection of highly pathogenic avian influenza virus H5N1 clade 2.3.4.4b in great skuas: a species of conservation concern in Great Britain. *Viruses* **14**, e212.

Bennett-Laso, B., Berazay, B., Munoz, G., Ariyama, N., Enciso, N., Braun, C., Kruger, L., Bartak, M., Gonzalez-Aravena, M. & Neira, V. (2024). Confirmation of highly pathogenic avian influenza H5N1 in skuas, Antarctica 2024. *Frontiers in Veterinary Science* **11**, e1423404.

Bennison, A., Adlard, S., Banyard, A. C., Blockley, F., Blyth, M., Browne, E., Day, G., Dunn, M. J., Falchieri, M., Fitzcharles, E., Forcada, J., Forster Davidson, J., Fox, A., Hall, R., Holmes, E., Hughes, K., James, J., Lynton-Jenkins, J., Marshall, S., McKenzie, D., Morley, S. A., Reid, S. M., Stubbs, I., Ratcliffe, N. & Phillips, R. A. (2024). A case study of highly pathogenic avian influenza (HPAI) H5N1 at Bird Island, South Georgia: the first documented outbreak in the Subantarctic region. *Bird Study* **71**, 380–391.

Beyit, A. D., Meki, I. K., Barry, Y., Haki, M. L., El Ghassem, A., Hamma, S. M., Abdelwahab, N., Doumbia, B., Ahmed Benane, H., Daf, D. S., Sidatt, Z. E. A., Ould Mekhalla, L., El Mamy, B., Gueya, M. O. B., Settypalli, T. B. K., Ouled Ahmed Ben Ali, H., Datta, S., Cattoli, G., Lamien, C. E. & Dundon, W. G. (2023). Avian influenza H5N1 in a great white pelican (*Pelecanus onocrotalus*), Mauritania 2022. *Veterinary Research Communications* **47**, 2193–2197.

Birkhead, T. R. & Hatchwell, B. J. (2025). The effect of the 2023 bird flu outbreak on the population biology of common guillemots on Skomer Island. *British Birds* **118**, 8–19.

Bregnballe, T., Herrmann, C., Globig, A., Günther, A., Staubach, C., Heise, J. N., Harder, T., Beer, M., Knief, U., Heinicke, T., Leivits, M., Lundström, K., Nurmoja, I., Liang, Y., Larsen, L. E., Hjulsager, C. K., Pohlmann, A. & Fox, A. D. (2024). Outbreaks of highly pathogenic avian influenza (HPAI) epidemics in Baltic great cormorant *Phalacrocorax carbo* colonies in 2021 and 2022. *Bird Study* **71**, 353–366.

BTO (British Trust for Ornithology) (2023). Avian influenza mortality rises in threatened gull and tern colonies, vol. 2025. British Trust for Ornithology, https://www.bto.org/community/news/202306-avian-influenza-mortality-rises-threatened-gull-and-tern-colonies.

Burke, B., Adcock, T., Boland, H., Büche, B., Fitzgerald, M., Johnson, G. C., Monaghan, J., Murray, T., Stubbings, E. & Newton, S. (2024). A case study of the 2023 highly pathogenic avian influenza (HPAI) outbreak in tern (Sternidae) colonies on the east coast of the Republic of Ireland. *Bird Study* **71**, 336–346.

Caliendo, V., Kleyheeg, E., Beerens, N., Camphuysen, K. C. J., Cazemier, R., Elbers, A. R. W., Fouchier, R. A. M., Kelder, L., Kuiken, T., Leopold, M., Slaterus, R., Spierenburg, M. A. H., van der Jeugd, H., Verdaat, H. & Rijks, J. (2024). Effect of 2020–21 and 2021–22 highly pathogenic avian influenza H5 epidemics on wild birds, the Netherlands. *Emerging Infectious Diseases* **30**, 50–57.

Camphuysen, K. & Gear, S. C. (2022). *Great skuas and northern gannets on Foula, summer 2022—an unprecedented, H5N1 related massacre*, V4 edition. NIOZ Royal Netherlands Institute for Sea Research, Texel, the Netherlands.

CapeNature (2025). Avian Influenza detected in Western Cape: public urged to stay alert, not alarmed, vol. 2026. CapeNature, https://www.capenature.co.za/news/2025/avian-influenza-detected-in-western-cape-public-urged-to-stay-alert-not-alarmed.

CBC Radio-Canada (2025*a*). Around 50 dead Canada geese found on frozen river in Lockport, Man., vol. 2026. CBC Radio-Canada, https://www.cbc.ca/news/canada/manitoba/dead-canada-goose-river-lockport-manitoba-9.7008431.

CBC Radio-Canada (2025*b*). Die-off of hundreds of birds something Manitoba hasn’t experienced ‘at this scale’, vol. 2026. CBC Radio-Canada, https://www.cbc.ca/news/canada/manitoba/dead-birds-carcasses-avian-influenza-9.7012752.

Clessin, A., Briand, F.-X., Tornos, J., Lejeune, M., Pasquale, C. D., Fischer, R., Souchaud, F., Hirchaud, E., Bralet, T., Guinet, C., McMahon, C. R., Grasland, B., Baele, G. & Boulinier, T. (2025). Circumpolar spread of avian influenza H5N1 to southern Indian Ocean islands. *Nature Communications* **16**, e8463.

Coffey, P. & Verspoor, R. S. (2025). Case study of the impact of an outbreak of high pathogenicity avian influenza (HPAI) on a seabird colony in Flintshire, Wales, United Kingdom. *Bird Study* **72**, 89–97.

Cormier, T. L., Barychka, T., Beaumont, M., Burt, T. V., English, M. D., Giacinti, J. A., Giroux, J.-F., Guillemette, M., Hargan, K. E., Jones, M., Lair, S., Lang, A. S., Lepage, C., Montevecchi, W. A., Rahman, I., Rail, J.-F., Robertson, G. J., Ronconi, R. A., Seyer, Y., Taylor, L. U., Ward, C. R. E., Wight, J., Wilhelm, S. I. & Avery-Gomm, S. (2024). Seabird and sea duck mortalities were lower during the second breeding season in eastern Canada following the introduction of highly pathogenic avian influenza A H5Nx viruses. *Bird Study* **71**, 367–379.

Cunningham, E. J. A., Amandine Gamble, A., Hart, T., Humphreys, E. M., Philip, E., Tyler, G. & Wood, M. J. (2022). The incursion of highly pathogenic avian influenza (HPAI) into North Atlantic seabird populations: an interim report from the 15th International Seabird Group conference. *Seabird* **34**, 67–73.

Cunningham, M. W., Brown, J., Hardman, R., Loerzel, S., Kluever, B. M., Zachariah, T. T., Donnelly, K. A., Poulson, R. L., Nemeth, N. M., Van Why, K., Sargent, R., Driscoll, C. P., Tegeler, A. K., Zhou, L., Guzman-Vargas, V., Lenoch, J., Ruder, M. G., Bommineni, Y. R. & Stallknecht, D. E. (2025). Outbreaks of highly pathogenic H5N1 influenza A virus infection in black vultures (*Coragyps atratus*), USA, 2022. *Journal of Wildlife Diseases* **61**, 955–966.

Daniels, B. L., Osnas, E. E., Boldenow, M. L., Gerlach, R. F., Ahlstrom, C. A., Coburn, S. M., Brook, M. J., Brubaker, M., Fischer, J. B., Koons, D. N., Matz, A. C., Murphy, M. A., Rizzolo, D. J., Scott, L. C., Sinnett, D. R., Thompson, J. M., Lenoch, J. B., Torchetti, M. K., Stallknecht, D. E., Poulson, R. L. & Ramey, A. M. (2025). Observational, virologic, and serologic data provide insights into an outbreak of highly pathogenic avian influenza among wild birds on the Yukon-Kuskokwim Delta, Alaska, USA, in 2022. *Journal of Wildlife Diseases* **61**, 1010–1027.

de Boer, P. (2024). *Broedvogels en broedsucces van Visdief en Noordse Stern op het broedeiland Stern in de Eems in 2023*. Sovon Vogelonderzoek Nederland, Nijmegen, the Netherlands.

de Lima, R. C., Estima, S. C., Tavares, M., Canabarro, P. L., Botta, S., Dias, L. A., da Silva, A. P., de Amorim, D. B. & de Oliveira, L. R. (2025). Impacts and lessons learned from the first highly pathogenic avian influenza (H5N1) outbreak in South American pinnipeds along the southern Brazilian coast. *Marine Mammal Science* **41**, e13163.

DFFE (Department of Forestry, Fisheries and the Environment) (2025). High pathogenicity avian influenza (H5N1) confirmed on Subantarctic Marion Island. Republic of South Africa, https://www.dffe.gov.za/mediarelease/h5n1_marionisland.

Djurdjević, B., Petrović, T., Gajdov, V., Vidanović, D., Vučićević, I., Samojlović, M. & Pajić, M. (2024). Natural infection of common cranes (*Grus grus*) with highly pathogenic avian influenza H5N1 in Serbia. *Frontiers in Veterinary Science* **11**, e1462546.

Duriez, O., Sassi, Y., Le Gall-Ladevèze, C., Giraud, L., Straughan, R., Dauverné, L., Terras, A., Boulinier, T., Choquet, R., Van De Wiele, A., Hirschinger, J., Guérin, J.-L. & Le Loc’h, G. (2023). Highly pathogenic avian influenza affects vultures’ movements and breeding output. *Current Biology* **33**, 3766–3774.

Duvenage, E. (2024). Avian flu suspected on South Africa’s remote Marion Island. *Nature Africa*. DOI: 10.1038/d44148-024-00345-x

EFSA (European Food Safety Authority), ECDC (European Centre for Disease Prevention and Control), EURL (European Union Reference Laboratory for Avian Influenza), Adlhoch, C., Fusaro, A., Gonzales, J. L., Kuiken, T., Marangon, S., Mirinaviciute, G., Niqueux, É., Stahl, K., Staubach, C., Terregino, C., Broglia, A. & Baldinelli, F. (2023*a*). Avian influenza overview December 2022 – March 2023. *EFSA Journal* **21**, e07917.

EFSA (European Food Safety Authority), ECDC (European Centre for Disease Prevention and Control), EURL (European Union Reference Laboratory for Avian Influenza), Adlhoch, C., Fusaro, A., Gonzales, J. L., Kuiken, T., Marangon, S., Niqueux, É., Staubach, C., Terregino, C., Aznar, I., Muñoz Guajardo, I. & Baldinelli, F. (2021). Avian influenza overview September – December 2021. *EFSA Journal* **19**, e07108.

EFSA (European Food Safety Authority), ECDC (European Centre for Disease Prevention and Control), EURL (European Union Reference Laboratory for Avian Influenza), Adlhoch, C., Fusaro, A., Gonzales, J. L., Kuiken, T., Marangon, S., Niqueux, É., Staubach, C., Terregino, C., Aznar, I., Muñoz Guajardo, I. & Baldinelli, F. (2022*a*). Avian influenza overview December 2021 – March 2022. *EFSA Journal* **20**, e07289.

EFSA (European Food Safety Authority), ECDC (European Centre for Disease Prevention and Control), EURL (European Union Reference Laboratory for Avian Influenza), Adlhoch, C., Fusaro, A., Gonzales, J. L., Kuiken, T., Marangon, S., Niqueux, É., Staubach, C., Terregino, C., Guajardo, I. M., Chuzhakina, K. & Baldinelli, F. (2022*b*). Avian influenza overview June – September 2022. *EFSA Journal* **20**, e07597.

EFSA (European Food Safety Authority), ECDC (European Centre for Disease Prevention and Control), EURL (European Union Reference Laboratory for Avian Influenza), Adlhoch, C., Fusaro, A., Gonzales, J. L., Kuiken, T., Melidou, A., Mirinavičiūtė, G., Niqueux, É., Ståhl, K., Staubach, C., Terregino, C., Baldinelli, F., Broglia, A. & Kohnle, L. (2023*b*). Avian influenza overview April – June 2023. *EFSA Journal* **21**, e08191.

EFSA (European Food Safety Authority), ECDC (European Centre for Disease Prevention and Control), EURL (European Union Reference Laboratory for Avian Influenza), Adlhoch, C., Fusaro, A., Gonzales, J. L., Kuiken, T., Mirinaviciute, G., Niqueux, É., Stahl, K., Staubach, C., Terregino, C., Broglia, A., Kohnle, L. & Baldinelli, F. (2023*c*). Avian influenza overview March – April 2023. *EFSA Journal* **21**, e08039.

EFSA (European Food Safety Authority), ECDC (European Centre for Disease Prevention and Control), EURL (European Union Reference Laboratory for Avian Influenza), Adlhoch, C., Fusaro, A., Gonzales, J. L., Kuiken, T., Mirinavičiūtė, G., Niqueux, É., Ståhl, K., Staubach, C., Terregino, C., Willgert, K., Baldinelli, F., Chuzhakina, K., Delacourt, R., Georganas, A., Georgiev, M. & Kohnle, L. (2023*d*). Avian influenza overview September – December 2023. *EFSA Journal* **21**, e8539.

EFSA (European Food Safety Authority), ECDC (European Centre for Disease Prevention and Control), EURL (European Union Reference Laboratory for Avian Influenza), Adlhoch, C., Fusaro, A., Gonzales, J. L., Kuiken, T., Mirinavičiūtė, G., Niqueux, É., Staubach, C., Terregino, C., Baldinelli, F., Rusinà, A. & Kohnle, L. (2023*e*). Avian influenza overview June – September 2023. *EFSA Journal* **21**, e08328.

EFSA (European Food Safety Authority), ECDC (European Centre for Disease Prevention and Control), EURL (European Union Reference Laboratory for Avian Influenza), Alexakis, L., Buczkowski, H., Ducatez, M., Fusaro, A., Gonzales, J. L., Kuiken, T., Ståhl, K., Staubach, C., Svartström, O., Terregino, C., Willgert, K., Delacourt, R. & Kohnle, L. (2024*a*). Avian influenza overview June – September 2024. *EFSA Journal* **22**, e9057.

EFSA (European Food Safety Authority), ECDC (European Centre for Disease Prevention and Control), EURL (European Union Reference Laboratory for Avian Influenza), Alexakis, L., Buczkowski, H., Ducatez, M., Fusaro, A., Gonzales, J. L., Kuiken, T., Ståhl, K., Staubach, C., Svartström, O., Terregino, C., Willgert, K., Melo, M. & Kohnle, L. (2025*a*). Avian influenza overview December 2024 – March 2025. *EFSA Journal* **23**, e9352.

EFSA (European Food Safety Authority), ECDC (European Centre for Disease Prevention and Control), EURL (European Union Reference Laboratory for Avian Influenza), Alexakis, L., Buczkowski, H., Ducatez, M., Fusaro, A., Gonzales, J. L., Kuiken, T., Ståhl, K., Staubach, C., Svartström, O., Terregino, C., Willgert, K., Melo, M. & Kohnle, L. (2025*b*). Avian influenza overview September – December 2024. *EFSA Journal* **23**, e9204.

EFSA (European Food Safety Authority), ECDC (European Centre for Disease Prevention and Control), EURL (European Union Reference Laboratory for Avian Influenza), Alexakis, L., Fusaro, A., Kuiken, T., Mirinavičiūtė, G., Ståhl, K., Staubach, C., Svartström, O., Terregino, C., Willgert, K., Delacourt, R., Goudjihounde, S. M., Grant, M., Tampach, S. & Kohnle, L. (2024*b*). Avian influenza overview March – June 2024. *EFSA Journal* **22**, e8930.

EFSA (European Food Safety Authority), ECDC (European Centre for Disease Prevention and Control), EURL (European Union Reference Laboratory for Avian Influenza), Fusaro, A., Gonzales, J. L., Kuiken, T., Mirinavičiūtė, G., Niqueux, É., Ståhl, K., Staubach, C., Svartström, O., Terregino, C., Willgert, K., Baldinelli, F., Delacourt, R., Georganas, A. & Kohnle, L. (2024*c*). Avian influenza overview December 2023 – March 2024. *EFSA Journal* **22**, e8754.

Else, R. & Watson, H. (2023). *Rathlin bird report 2021–2022*. https://rathlinstickybeak.wordpress.com/wp-content/uploads/2023/02/rathlin-bird-report-2021-2022.pdf

Esaki, M., Okuya, K., Tokorozaki, K., Haraguchi, Y., Hasegawa, T. & Ozawa, M. (2025). Highly pathogenic avian influenza A(H5N1) outbreak in endangered cranes, Izumi Plain, Japan, 2022–23. *Emerging Infectious Diseases* **31**, 937–947.

Falchieri, M., Bentley, E., Coombes, H. A., Mollett, B. C., Terrey, J., Holland, S., Stubbings, E., Mcginn, N., Cooper, J., Ahmad, S., Lewis, J., Clifton, B., Collinson, N., Aegerter, J., Venkatesh, D., Russell, D. J. F., James, J., Reid, S. M. & Banyard, A. C. (2026). Detection of two high pathogenicity avian influenza virus (HPAIV) subtypes, H5N1 and H5N5, in a mass mortality event in wild seabirds and co-location with dead seals. *Transboundary and Emerging Diseases* **2026**, e4680980.

Falkland Islands Department of Agriculture (2025). Suspect and confirmed cases of avian influenza in the Falkland Islands, vol. 2025. Falkland Islands Government, https://falklands.gov.fk/agriculture/avian-influenza.

Gamarra-Toledo, V., Plaza, P. I., Angulo, F., Gutiérrez, R., García-Tello, O., Saravia-Guevara, P., Mejía-Vargas, F., Epiquién-Rivera, M., Quiroz-Jiménez, G., Martinez, P., Huamán-Mendoza, D., Inga-Díaz, G., La Madrid, L. E., Luyo, P., Ventura, S. & Lambertucci, S. A. (2023). Highly pathogenic avian influenza (HPAI) strongly impacts wild birds in Peru. *Biological Conservation* **286**, e110272.

Giacinti, J. A., Signore, A. V., Jones, M. E. B., Bourque, L., Lair, S., Jardine, C., Stevens, B., Bollinger, T., Goldsmith, D., British Columbia Wildlife, A. I. V. S. P., Pybus, M., Stasiak, I., Davis, R., Pople, N., Nituch, L., Brook, R. W., Ojkic, D., Masse, A., Dimitri-Masson, G., Parsons, G. J., Baker, M., Yason, C., Harms, J., Jutha, N., Neely, J., Berhane, Y., Lung, O., French, S. K., Myers, L., Provencher, J. F., Avery-Gomm, S., Robertson, G. J., Barychka, T., Gurney, K. E. B., Wight, J., Rahman, I., Hargan, K., Lang, A. S., Montevecchi, W. A., Burt, T. V., Brown, M. G. C., Pekarik, C., Thompson, T., McLaughlin, A., Willie, M., Wilson, L., Flemming, S. A., Ross, M. V., Leafloor, J., Baldwin, F., Sharp, C., Lewis, H., Beaumont, M., Hanson, A., Ronconi, R. A., Reed, E., Campbell, M., Saunders, M. & Soos, C. (2024). Avian influenza viruses in wild birds in Canada following incursions of highly pathogenic H5N1 virus from Eurasia in 2021–2022. *mBio* **15**, e0320323.

Giralt Paradell, O., Goh, T., Popov, D., Rogan, E. & Jessopp, M. (2023). Estimated mortality of the highly pathogenic avian influenza pandemic on northern gannets (*Morus bassanus*) in southwest Ireland. *Biology Letters* **19**, e20230090.

Glazunova, A., Krasnova, E., Bespalova, T., Sevskikh, T., Lunina, D., Titov, I., Sindryakova, I. & Blokhin, A. (2024). A highly pathogenic avian influenza virus H5N1 clade 2.3.4.4 detected in Samara Oblast, Russian Federation. *Frontiers in Veterinary Science* **11**, e1244430.

Gopinathan, L. (2024). Sitting ducks: how likely is a bird flu pandemic in India?, vol. 2025. Gavi, The Vaccine Alliance, https://www.gavi.org/vaccineswork/sitting-ducks-how-likely-bird-flu-pandemic-india.

Graham, L. (2022). Bird flu has killed nearly 1,500 threatened Caspian terns on Lake Michigan islands, vol. 2025. Great Lakes Now, https://www.michiganpublic.org/environment-climate-change/2022-06-29/bird-flu-has-killed-nearly-1-500-threatened-caspian-terns-on-lake-michigan-islands.

Grémillet, D., Ponchon, A., Provost, P., Gamble, A., Abed-Zahar, M., Bernard, A., Courbin, N., Delavaud, G., Deniau, A., Fort, J., Hamer, K. C., Jeavons, R., Lane, J. V., Langley, L., Matthiopoulos, J., Poupart, T., Prudor, A., Stephens, N., Trevail, A., Wanless, S., Votier, S. C. & Jeglinski, J. W. E. (2023). Strong breeding colony fidelity in northern gannets following high pathogenicity avian influenza virus (HPAIV) outbreak. *Biological Conservation* **286**, e110269.

Griffin, L. R. & Peach, W. J. (2024). Impacts of highly pathogenic avian influenza (HPAI) on a barnacle goose *Branta leucopsis* population wintering on the Solway Firth, UK. *Bird Study* **71**, 392–403.

Gulati, V. (2021). Bird flu: bar-headed geese worst hit in Pong wintering ground, vol. 2025. Daijiworld News Network, https://daijiworld.com/news/newsDisplay?newsID=789836.

Günther, A., Herrmann, C., Sehl-Ewert, J., Piro, S., Ahrens, A. K., Calvelage, S., Pohlmann, A., Beer, M. & Harder, T. (2026). Highly pathogenic avian influenza A(H5N1) clade 2.3.4.4b virus and mass mortality in Eurasian cranes, Germany, 2025. *Emerging Infectious Diseases* **32**, DOI: 10.3201/eid3205.260170.

Günther, A., Krone, O., Globig, A., Pohlmann, A., King, J., Fast, C., Grund, C., Hennig, C., Herrmann, C., Piro, S., Rubbenstroth, D., Schulz, J., Staubach, C., Stacker, L., Ulrich, L., Ziegler, U., Harder, T. & Beer, M. (2024). Avian raptors are indicator species and victims of high pathogenicity avian influenza virus HPAIV H5N1 (clade 2.3.4.4b) in Germany. *Scientific Reports* **14**, e28779.

Haman, K. H., Pearson, S. F., Brown, J., Frisbie, L. A., Penhallegon, S., Falghoush, A. M., Wolking, R. M., Torrevillas, B. K., Taylor, K. R., Snekvik, K. R., Tanedo, S. A., Keren, I. N., Ashley, E. A., Clark, C. T., Lambourn, D. M., Eckstrand, C. D., Edmonds, S. E., Rovani-Rhoades, E. R., Oltean, H., Wilkinson, K., Fauquier, D., Black, A. & Waltzek, T. B. (2024). A comprehensive epidemiological approach documenting an outbreak of H5N1 highly pathogenic avian influenza virus clade 2.3.4.4b among gulls, terns, and harbor seals in the Northeastern Pacific. *Frontiers in Veterinary Science* **11**, e1483922.

Harris, M. P. & Wanless, S. (2023). A major mortality of gannets due to a probable outbreak of high pathogenicity avian influenza on Sule Skerry in 2022. *Scottish Birds* **43**, 228–231.

Hjulsager, C. K., Liang, Y., Thorup, C., Larsen, L. E., Jensen, T. K., Breum, S. & Bregnballe, T. (2025). Mass mortalities caused by different genotypes of HPAIV H5N1 clade 2.3.4.4b in colony breeding black-headed gulls and Sandwich terns in Denmark 2022 and 2023. *Canadian Journal of Microbiology* **72**, 1–10.

Homma, H. (2025). Bird flu suspected in mass deaths of sea animals in eastern Hokkaido. In *The Mainichi*, vol. May 8, 2025. Accessed December 29, 2025, https://mainichi.jp/english/articles/20250507/p2a/00m/0li/020000c.

Iervolino, M., Günther, A., Begeman, L., Aguado, B., Bestebroer, T. M., Bellido-Martin, B., Coerper, A., Fornillo, M. V., Fusaro, B., Ibañez, A. E., Leijten, L., Lisovski, S., Mañez, M. B., Reade, A., van Run, P., Soto, F., Wallis, B., Dewar, M., Alcamí, A., Beer, M., Vanstreels, R. E. T. & Kuiken, T. (2026). The expanding H5N1 avian influenza panzootic causes high mortality of skuas in Antarctica. *Scientific Reports* **16**, e4604.

Indykiewicz, P., Przymencki, M., Minias, P., Jakubas, D., Litwiniak, K., Zieliński, P., Janiszewski, T., Włodarczyk, R., Ledwoń, M., Nowakowski, J., Dulisz, B., Domańska-Blicharz, K., Świętoń, E., Śmietanka, K., Bukaciński, D., Bukacińska, M., Buczyński, A., Beuch, S., Chodkiewicz, T., Betleja, J., Bzoma, S., Marchowski, D., Sikora, A., Bednarz, Ł., Antczak, J., Dylik, A., Kajzer, Z., Król, W., Krajewski, Ł., Menderski, S., Rapczyński, J., Rubacha, S., Szczerbik, R., Szymczak, J., Walasz, K. & Wylegała, P. (2025). Impact of highly pathogenic avian influenza virus (HPAIV) on black-headed gulls *Chroicocephalus ridibundus* population in Poland in 2023. *Avian Pathology* **54**, 498–511.

Jatta, D., Wille, M., Ballmann, M. Z., Youm, B. N., Camara, F., Dodman, T. & Fox, O. J. L. (2025). Outbreak of high pathogenicity avian influenza (H5N1) in seabird populations of West Africa. *Bird Study* **72**, 110–120.

Ke, X., Yao, Z., Tang, Y., Yang, M., Li, Y., Yang, G., Chen, J., Chen, G., Feng, W., Zheng, H. & Chen, Q. (2022). Highly pathogenic avian influenza A (H5N1) virus in swans, Central China, 2021. *Microbiology Spectrum* **10**, e02315-22.

King, J., Pohlmann, A., Bange, A., Horn, E., Hälterlein, B., Breithaupt, A., Globig, A., Günther, A., Kelm, A., Wiedemann, C., Grund, C., Haecker, K., Garthe, S., Harder, T., Beer, M. & Schwemmer, P. (2024). Red knots in Europe: a dead end host species or a new niche for highly pathogenic avian influenza? *Journal of General Virology* **105**, e002003.

Knief, U., Ahrens, A. K., Allendorf, V., Behringer, C. J., Bertram, J., Bouwhuis, S., Fiedler, W., Globig, A., Günther, A., Herrmann, C., Knauf, S., Marchowski, D., Piro, S., Pohlmann, A., Rollins, R. E., Staubach, C. & Harder, T. (2026). Outbreak dynamics of high pathogenicity avian influenza virus H5N1, clade 2.3.4.4b euBB, in black-headed gulls and common terns in Germany in 2023. *Avian Pathology*, [DOI: 10.1080/03079457.2026.2659834](https://doi.org/10.1080/03079457.2026.2659834).

Knief, U., Bregnballe, T., Alfarwi, I., Ballmann, M. Z., Brenninkmeijer, A., Bzoma, S., Chabrolle, A., Dimmlich, J., Engel, E., Fijn, R., Fischer, K., Hälterlein, B., Haupt, M., Hennig, V., Herrmann, C., in ‘t Veld, R., Kirchhoff, E., Kristersson, M., Kühn, S., Larsson, K., Larsson, R., Lawton, N., Leopold, M., Lilipaly, S., Lock, L., Marty, R., Matheve, H., Meissner, W., Morrison, P., Newton, S., Olofsson, P., Packmor, F., Pedersen, K. T., Redfern, C., Scarton, F., Schenk, F., Scher, O., Serra, L., Sibille, A., Smith, J., Smith, W., Sterup, J., Stienen, E., Strassner, V., Valle, R. G., van Bemmelen, R. S. A., Veen, J., Vervaeke, M., Weston, E., Wojcieszek, M. & Courtens, W. (2024). Highly pathogenic avian influenza causes mass mortality in Sandwich tern *Thalasseus sandvicensis* breeding colonies across north-western Europe. *Bird Conservation International* **34**, e6.

Kuiken, T., Vanstreels, R. E. T., Banyard, A., Begeman, L., Breed, A. C., Dewar, M., Fijn, R., Serafini, P. P., Uhart, M. & Wille, M. (2026). Emergence, spread, and impact of high-pathogenicity avian influenza H5 in wild birds and mammals of South America and Antarctica. *Conservation Biology* **40**, e70052.

Kydyrmanov, A., Karamendin, K., Kassymbekov, Y., Daulbayeva, K., Sabyrzhan, T., Khan, Y., Nuralibekov, S., Baikara, B. & Fereidouni, S. (2024). Mass mortality in terns and gulls associated with highly pathogenic avian influenza viruses in Caspian Sea, Kazakhstan. *Viruses* **16**, e1661.

Lane, J. V., Jeglinski, J. W. E., Avery-Gomm, S., Ballstaedt, E., Banyard, A. C., Barychka, T., Brown, I. H., Brugger, B., Burt, T. V., Careen, N., Castenschiold, J. H. F., Christensen-Dalsgaard, S., Clifford, S., Collins, S. M., Cunningham, E., Danielsen, J., Daunt, F., D'entremont, K. J. N., Doiron, P., Duffy, S., English, M. D., Falchieri, M., Giacinti, J., Gjerset, B., Granstad, S., Grémillet, D., Guillemette, M., Hallgrímsson, G. T., Hamer, K. C., Hammer, S., Harrison, K., Hart, J. D., Hatsell, C., Humpidge, R., James, J., Jenkinson, A., Jessopp, M., Jones, M. E. B., Lair, S., Lewis, T., Malinowska, A. A., McCluskie, A., McPhail, G., Moe, B., Montevecchi, W. A., Morgan, G., Nichol, C., Nisbet, C., Olsen, B., Provencher, J., Provost, P., Purdie, A., Rail, J.-F., Robertson, G., Seyer, Y., Sheddan, M., Soos, C., Stephens, N., Strøm, H., Svansson, V., Tierney, T. D., Tyler, G., Wade, T., Wanless, S., Ward, C. R. E., Wilhelm, S. I., Wischnewski, S., Wright, L. J., Zonfrillo, B., Matthiopoulos, J. & Votier, S. C. (2024). High pathogenicity avian influenza (H5N1) in northern gannets (*Morus bassanus*): global spread, clinical signs and demographic consequences. *Ibis* **166**, 633–650.

León, F., Ulloa-Contreras, C., Pizarro, E. J., Castillo-Torres, P. N., Díaz-Morales, K. B., Franco, A. C., Timm, F. C. B., Corrêa, M. L., Krüger, L., Poulin, E., Pardo-Roa, C. & Vianna, J. A. (2025). Skuas mortalities linked to positives HPAIV A/H5 beyond Polar Antarctic Circle. *bioRxiv* 2025.03.02.640960.

Lo, F., Zecchin, B., Diallo, A., Racky, O., Tassoni, L., Diop, A., Diouf, M., Diouf, M., Samb, Y., Pastori, A., Gobbo, F., Ellero, F., Diop, M., Lo, M., Diouf, M., Fall, M., Ndiaye, A., Gaye, A., Badiane, M., Lo, M., Youm, B., Ndao, I., Niaga, M., Terregino, C., Diop, B., Ndiaye, Y., Angot, A., Seck, I., Niang, M., Soumare, B., Fusaro, A. & Monne, I. (2022). Intercontinental spread of Eurasian highly pathogenic avian influenza A(H5N1) to Senegal. *Emerging Infectious Diseases* **28**, 234–237.

Lublin, A., Shkoda, I., Simanov, L., Hadas, R., Berkowitz, A., Lapin, K., Farnoushi, Y., Katz, R., Nagar, S., Kharboush, C., Peri Markovich, M. & King, R. (2023). The history of highly-pathogenic avian influenza in Israel (H5-subtypes): from 2006 to 2023. *Israel Journal of Veterinary Medicine* **78**, 13–26.

LVportals.lv (2023). Highly pathogenic avian influenza H5N1 for dead wild birds in Kraslava has also been confirmed, vol. 2025. LVportals.lv, https://lvportals.lv/dienaskartiba/351603-ari-kraslava-mirusajiem-savvalas-putniem-apstiprinata-augsti-patogena-putnu-gripa-h5n1-2023.

Marchowski, D., Chara, P., Borek, Ł., Kajzer, Z. & Bzoma, S. (2024). Effects of highly pathogenic avian influenza among colonial waterbirds in the lower Odra valley. *Acta Ornithologica* **59**, 23–33.

Martínez, R., Agulló-Ros, I., Gonzálvez, M., Ruano, M. J., Zecchin, B., Zorrilla, I., Guerra, R., Paniagua, J., Cano-Terriza, D. & García-Bocanegra, I. (2025). Highly pathogenic avian influenza A (H5N1) infection in bearded vultures in Spain. *Zoonoses and Public Health* **72**, 544–549.

Ministry of Agriculture, Fisheries, Water and Land Reform (2025). Outbreak of highly pathogenic avian influenza (H5N1) in wild birds in Karas region around Lüderitz. Republic of Namibia.

Molini, U., Yabe, J., Meki, I. K., Ouled, H. A. B. A., Settypalli, T. B. K., Datta, S., Coetzee, L. M., Hamunyela, E., Khaiseb, S., Cattoli, G., Lamien, C. E. & Dundon, W. G. (2023). Highly pathogenic avian influenza H5N1 virus outbreak among cape cormorants (*Phalacrocorax capensis*) in Namibia, 2022. *Emerging Microbes & Infections* **12**, e2167610.

Moutinho, S. (2025). Deadly avian flu strain is spreading rapidly in Antarctica. *Science* **387**, 1130–1131.

Muñoz, G., Ulloa, M., Alegría, R., Quezada, B., Bennett, B., Enciso, N., Atavales, J., Johow, M., Aguayo, C., Araya, H. & Neira, V. (2024). Stranding and mass mortality in Humboldt penguins (*Spheniscus humboldti*), associated to HPAIV H5N1 outbreak in Chile. *Preventive Veterinary Medicine* **227**, e106206.

Nadler-Valency, R., Lourie, E. & Sela-Klein, D. (2025). Avian influenza (H5N1) outbreak report in wild birds – Hula Valley, Israel: a comparison of two outbreaks (2021/22 and 2024). *Israel Journal of Veterinary Medicine* **80**, 3–13.

Nagy, A., Černíková, L. & Sedlák, K. (2025). Genetic data and meteorological conditions suggesting windborne transmission of H5N1 high-pathogenicity avian influenza between commercial poultry outbreaks. *PLoS One* **20**, e0319880.

NatureScot (2023*a*). Avian flu causes another challenging summer for seabirds, vol. 2025. NatureScot, https://www.nature.scot/avian-flu-causes-another-challenging-summer-seabirds.

NatureScot (2023*b*). NatureScot scientific advisory committee sub-group on avian influenza report on the H5N1 outbreak in wild birds 2020-2023, vol. 2025. NatureScot, https://www.nature.scot/doc/naturescot-scientific-advisory-committee-sub-group-avian-influenza-report-h5n1-outbreak-wild-birds.

Ndao, C. B. (2023). Response to the outbreak of avian flu in Senegal, Gambia, and Guinea-Bissau, vol. 2025. BirdLife International, https://www.birdlife.org/news/2023/05/24/response-to-the-outbreak-of-avian-flu-in-senegal-gambia-and-guinea-bissau/.

News24 (2021). Bird flu behind mass pelican deaths in Senegal, vol. 02/04/2025. News24, https://www.news24.com/bird-flu-behind-mass-pelican-deaths-in-senegal-20210130.

Ogrzewalska, M., Pereira, E. C., Vanstreels, R. E. T., Campista, E., Correa Junior, L., Macedo, L., Appolinario, L. R., Brandão, M. L., Vilela, R., Degrave, W., Motta, F. C., Siqueira, M. M. & Resende, P. C. (2024). High pathogenicity avian influenza virus (HPAIV) H5N1 clade 2.3.4.4b recovered from a kelp gull (*Larus dominicanus*) in the South Shetland Islands, Antarctica. *bioRxiv* 2024.12.29.630510.

Owens, R. (2024). HPAI in Northern Ireland seabirds 2023. In *The Northern Ireland Seabird Report 2023*. (eds K. J. Booth and H. El Haddad), pp. 64–70. British Trust for Ornithology, Thetford, UK.

Pardo-Roa, C., Nelson, M. I., Ariyama, N., Aguayo, C., Almonacid, L. I., Gonzalez-Reiche, A. S., Muñoz, G., Ulloa, M., Ávila, C., Navarro, C., Reyes, R., Castillo-Torres, P. N., Mathieu, C., Vergara, R., González, Á., González, C. G., Araya, H., Castillo, A., Torres, J. C., Covarrubias, P., Bustos, P., van Bakel, H., Fernández, J., Fasce, R. A., Johow, M., Neira, V. & Medina, R. A. (2025). Cross-species and mammal-to-mammal transmission of clade 2.3.4.4b highly pathogenic avian influenza A/H5N1 with PB2 adaptations. *Nature Communications* **16**, e2232.

Perlman, N. I. Y. (2024). Avian influenza outbreak in Israel in winter 2024, vol. 2025. BirdLife Israel, https://www.birds.org.il/en/article/nadav-israeli-and-yoav-perlman-avian-influenza-march-2024.

Pohlmann, A., Stejskal, O., King, J., Bouwhuis, S., Packmor, F., Ballstaedt, E., Hälterlein, B., Hennig, V., Stacker, L., Graaf, A., Hennig, C., Günther, A., Liang, Y., Hjulsager, C., Beer, M. & Harder, T. (2023). Mass mortality among colony-breeding seabirds in the German Wadden Sea in 2022 due to distinct genotypes of HPAIV H5N1 clade 2.3.4.4b. *Journal of General Virology* **104**, e001834.

Przymencki, M., Beuch, S., Indykiewicz, P., Litwiniak, K., Bukaciński, D., Bukacińska, M., Zieliński, P., Betleja, J., Marchowski, D., Ledwoń, M., Bzoma, S., Buczyński, A., Wardecki, Ł., Bednarz, Ł., Flis, A., Łożyńska, H., Kusal, B. & Chodkiewicz, T. (2024). Mortality among scarce breeding gulls and terns during a highly pathogenic avian influenza (HPAI) H5N1 virus outbreak in Poland during 2023. *Bird Study* **71**, 326–335.

Reuters (2024). Bird flu found in western China as US combats cattle outbreak, vol. 2025. Reuters, https://www.reuters.com/world/china/bird-flu-found-western-china-us-combats-cattle-outbreak-2024-05-18/.

Reuters (2025). Bird flu likely killed hundreds of seal pups on Sub-Antarctic island, vol. 2026. Reuters, https://www.reuters.com/business/environment/australia-says-h5-bird-flu-suspected-sub-antarctic-island-2025-10-23/.

Romero, M. A., Bartés, S. N., Grandi, M. F., Brogger, M., Svendsen, G., González, R. & Crespo, E. A. (2025). Impact of the avian influenza outbreak on the population dynamics of South American sea lions *Otaria byronia* in northern Patagonia. *Marine Mammal Science* **41**, e70031.

Rømo, G., Åkesson, C. P., Reiertsen, T. K., Fosse, J. H., Bøe, C. A., Austbø, L., Åkerstedt, J., Saghafian, M., Helberg, M., Hungnes, O., Gjerset, B., Granstad, S., Hogseth, G., Løtvedt, S., Døsen, A. & Tønnessen, R. (2026). Highly pathogenic avian influenza A (H5N1) caused mass death among black-legged kittiwakes (*Rissa tridactyla*) in Norway, 2023. *Transboundary and Emerging Diseases* **2026**, e2963364.

Ross, C. S., Byrne, A. M. P., Mahmood, S., Thomas, S., Reid, S., Freath, L., Griffin, L. R., Falchieri, M., Holmes, P., Goldsmith, N., Shaw, J. M., MacGugan, A., Aegerter, J., Hansen, R., Brown, I. H. & Banyard, A. C. (2024). Genetic analysis of H5N1 high-pathogenicity avian influenza virus following a mass mortality event in wild geese on the Solway Firth. *Pathogens* **13**, e83.

Royal HaskoningDHV (2023). *Sheringham Shoal and Dudgeon offshore wind farm extension projects—review of 2022 highly pathogenic avian influenza (HPAI) outbreak on relevant UK seabird colonies*. equinor.

Seo, Y.-R., Lee, S.-H., Jeong, S., Cho, H., Kim, D., Kim, D.-J., Si, Y.-J., Jeong, H., Lee, S., Song, C.-S., Swayne, D. E. & Lee, D.-H. (2024). Genetic and pathological analysis of hooded cranes (*Grus monacha*) naturally infected with clade 2.3.4.4b highly pathogenic avian influenza H5N1 virus in South Korea in the winter of 2022. *Frontiers in Veterinary Science* **11**, e1499440.

SERNAPESCA (2024). Influenza Aviar, vol. 2025. Servicio Nacional de Pesca y Acuicultura, https://www.sernapesca.cl/influenza-aviar/.

Sobolev, I., Alekseev, A., Sharshov, K., Chistyaeva, M., Ivanov, A., Kurskaya, O., Ohlopkova, O., Moshkin, A., Derko, A., Loginova, A., Solomatina, M., Gadzhiev, A., Bi, Y. & Shestopalov, A. (2024). Highly pathogenic avian influenza A(H5N1) virus clade 2.3.4.4b infections in seals, Russia, 2023. *Emerging Infectious Diseases* **30**, 2160–2164.

Sobolev, I., Gadzhiev, A., Sharshov, K., Ohlopkova, O., Stolbunova, K., Fadeev, A., Dubovitskiy, N., Glushchenko, A., Irza, V., Perkovsky, M., Litvinov, K., Meshcheriakova, N., Petherbridge, G. & Shestopalov, A. (2023). Highly pathogenic avian influenza A(H5N1) virus-induced mass death of wild birds, Caspian Sea, Russia, 2022. *Emerging Infectious Diseases* **29**, 2528–2532.

Steinfurth, A., Lynton-Jenkins, J. G., Cleeland, J., Mollett, B. C., Coombes, H. A., Moores, A., Neal, R., Clifton, B., Falchieri, M., Jones, C. W., Risi, M. M., Gold, S., James, J., Ryan, P. G., González-Solís, J. & Banyard, A. C. (2026). Investigating high pathogenicity avian influenza virus incursions to remote islands: detection of H5N1 on Gough Island in the South Atlantic Ocean. *Emerging Microbes & Infections* **15**, e2627076.

Stokstad, E. (2022). Deadly flu spreads through North American birds. *Science* **376**, 441–442.

Sueur, F. (2023). Impact of highly pathogenic H5N1 avian influenza on some gull populations on the coast of Hauts-de-France (France). In *Birds - Conservation, Research and Ecology*. (ed H. Mikkola). IntechOpen, Rijeka.

Sultankulova, K. T., Argimbayeva, T. U., Aubakir, N. A., Bopi, A., Omarova, Z. D., Melisbek, A. M., Karamendin, K., Kydyrmanov, A., Chervyakova, O. V., Kerimbayev, A. A., Burashev, Y. D., Kasymbekov, Y. T. & Orynbayev, M. B. (2024). Reassortants of the highly pathogenic influenza virus A/H5N1 causing mass swan mortality in Kazakhstan from 2023 to 2024. *Animals* **14**, e3211.

Szteren, D. & Franco-Trecu, V. (2024). Incidence of highly pathogenic avian influenza H5N1 in pinnipeds in Uruguay. *Diseases of Aquatic Organisms* **160**, 65–74.

Tarasov, V. V. (2022). A sharp decrease in the number of Dalmatian pelican *Pelecanus crispus* (Aves: Pelecaniformes) in the Urals and Western Siberia in 2021. *Tomsk State University Journal of Biology* 128–146.

Taylor, L., Ronconi, R., Spina, H., Jones, M., Ogbunugafor, C. & Ayala, A. (2023). Limited outbreak of highly pathogenic influenza A(H5N1) in herring gull colony, Canada, 2022. *Emerging Infectious Diseases* **29**, 2150–2154.

Towe, R., Latimer, J., Barron, P., Doyle, H. & Burke, B. (2025). *Lady’s Island Lake tern conservation report 2025*. BirdWatch Ireland Seabird Conservation Report, Kilcoole, Wicklow.

Tremlett, C. J., Cleasby, I. R., Bolton, M. & Wilson, L. J. (2024). Declines in UK breeding populations of seabird species of conservation concern following the outbreak of high pathogenicity avian influenza (HPAI) in 2021–2022. *Bird Study* **71**, 293–310.

Uhart, M. M. & Vanstreels, R. E. T. (2025). Overview of high pathogenicity avian influenza H5N1 clade 2.3.4.4b in wildlife from Central and South America, October 2022–September 2025. *Canadian Journal of Microbiology* **71**, 1–8.

Uhart, M. M., Vanstreels, R. E. T., Nelson, M. I., Olivera, V., Campagna, J., Zavattieri, V., Lemey, P., Campagna, C., Falabella, V. & Rimondi, A. (2024). Epidemiological data of an influenza A/H5N1 outbreak in elephant seals in Argentina indicates mammal-to-mammal transmission. *Nature Communications* **15**, e9516.

USGS-WHISPers (Wildlife Health Information Sharing Partnership) (2025). ‘Mortality/Morbidity’ caused by ‘Highly Pathogenic Avian Influenza (AI virus H5)’, ‘Highly Pathogenic Avian Influenza (AI virus H5N1)’, ‘Highly Pathogenic Avian Influenza (AI virus, not otherwise specified)’ in ‘Bird’ and ‘Mammal’ from ‘1/1/2021 – 12/31/2025’, vol. 2026. Wildlife Health Information Sharing Partnership, https://whispers.usgs.gov/home.

Valle, R. G., Carretta, G., Zelco, S., Selle, V. & Scarton, F. (2024). H5N1 avian influenza outbreak caused massive mortality of chicks of Sandwich tern *Thalasseus sandvicensis* in the Lagoon of Venice. *Ardea* **112**, 239–246.

Wood, A. (2025). North Cave Wetlands: nine mute swans die in avian flu outbreak, vol. 2025. The Yorkshire Post, https://www.yorkshirepost.co.uk/news/north-cave-wetlands-nine-mute-swans-die-in-avian-flu-outbreak-4978710.
